# Designing plant–transparent agrivoltaics

**DOI:** 10.1038/s41598-023-28484-5

**Published:** 2023-02-02

**Authors:** Eric J. Stallknecht, Christopher K. Herrera, Chenchen Yang, Isaac King, Thomas D. Sharkey, Richard R. Lunt, Erik S. Runkle

**Affiliations:** 1 Department of Horticulture, Michigan State University, East Lansing, MI USA; 2 Department of Chemical Engineering and Materials Science, Michigan State University, East Lansing, MI USA; 3 MSU-DOE Plant Research Laboratory and Department of Biochemistry and Molecular Biology, Michigan State University, East Lansing, MI USA

**Keywords:** Light responses, Plant sciences, Energy science and technology, Renewable energy

## Abstract

Covering greenhouses and agricultural fields with photovoltaics has the potential to create multipurpose agricultural systems that generate revenue through conventional crop production as well as sustainable electrical energy. In this work, we evaluate the effects of wavelength-selective cutoffs of visible and near-infrared (biologically active) radiation using transparent photovoltaic (TPV) absorbers on the growth of three diverse, representative, and economically important crops: petunia, basil, and tomato. Despite the differences in TPV harvester absorption spectra, photon transmission of photosynthetically active radiation (PAR; 400–700 nm) is the most dominant predictor of crop yield and quality. This indicates that different wavebands of blue, red, and green are essentially equally important to these plants. When the average photosynthetic daily light integral is > 12 mol m^–2^ d^–1^, basil and petunia yield and quality is acceptable for commercial production. However, even modest decreases in TPV transmission of PAR reduces tomato growth and fruit yield. These results identify crop-specific design requirements that exist for TPV harvester transmission and the necessity to maximize transmission of PAR to create the most broadly applicable TPV greenhouse harvesters for diverse crops and geographic locations. We determine that the deployment of 10% power conversion efficiency (*PCE*) plant-optimized TPVs over approximately 10% of total agricultural and pasture land in the U.S. would generate 7 TW, nearly double the entire energy demand of the U.S.

## Introduction

**Figure 1:**
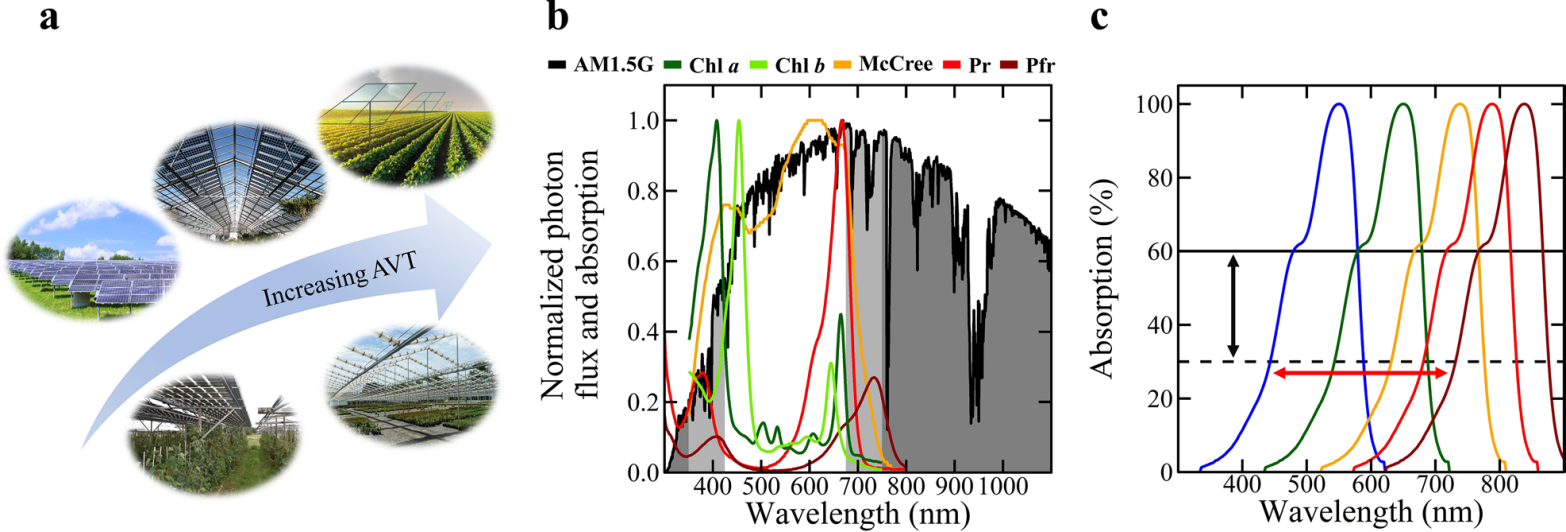
Conceptualization of key agrivoltaic principles. (**a**) Ideally, agrivoltaic systems would have high transmission of photosynthetically active radiation (PAR; photons between 400 and 700 nm). The simplest implementation of agrivoltaics is by deploying opaque modules over or adjacent to crops. Higher average photosynthetic transmittance (*APT*) can be achieved by moving to wavelength-selective modules that do not absorb PAR. Image credit (clockwise starting from the left-most picture): “Poultry Field Day Solar Panel” by Delaware Cooperative Extension (via creativecommons.org); “Beaulieu Abbey, Palace & Gardens 22-09-2012” by Karen Roe (via creativecommons.org), modified to add representative solar cells; “Garage of Green Furrows” by Ian Sane (via creativecommons.org), modified to add representative TPV modules over the field; “20120625-OSEC-RH-0019” by USDAgov (via creativecommons.org); credit: Groen Leven (via hortidaily.com). (**b**) Having a high PAR transmission is important because plants use photons from this waveband for photosynthesis and as signals for photomorphogenesis. To characterize this window, we have plotted the absorption spectra of chlorophyll (Chl) *a* and *b*, red-absorbing phytochrome (Pr) and far-red-absorbing phytochrome (Pfr), and the averaged quantum yield (mol CO_2_ fixed per mol photon absorbed as a function of wavelength; denoted as McCree) of many plants^6^. Absorbing visible radiation (VIS) would be energetically advantageous for building-integrated PV (BIPV) panels but could negatively influence greenhouse crop growth and development. (**c**) Thus, in this research we look at plant growth and productivity as a function of cutoffs of VIS and near infra-red wavebands and of overall transmission using neutral-density treatments.

The incorporation of photovoltaics (PV) into agriculture has drawn significant interest recently to address increased food insecurity and energy demand^1^. Agrivoltaics is the utilization of sunlight for both plant production and solar energy harvesting^2,3^. These two fields are often seen as competitive rather than cooperative because they can both occupy large areas of land to maximize sunlight utilization. Indeed, most agrivoltaic-based efforts have looked to integrate opaque solar panels (such as Si modules) over and around agricultural spaces so that there is often a strong tradeoff between allowing light to penetrate to the plants or be utilized for solar electricity generation. Despite this, photovoltaics have potential synergistic benefits with plant production. In addition to generating electricity to power greenhouse load (e.g., lights, fans, and other equipment), PV modules can also decrease water consumption by reducing the rate of evaporation from soil and transpiration from plants^4,5^. Both plant responses and PV power generation are key considerations in designing agrivoltaic systems. One way to overcome the severe limitation of opaque agrivoltaics is to design new PVs that can maintain plant yield and quality by minimizing PV impact on transmission of photons with wavelengths between 400 and 700 nm, which is referred to as photosynthetically active radiation (PAR). Figure 1a illustrates the progression and outlook of agrivoltaic approaches, including current approaches shown here, based on increasing transparency, starting with opaque PV modules, and moving towards PAR-transparent PV devices.

Plant productivity typically increases with photon flux density of PAR^7–12^. This is measured instantaneously as the photosynthetic photon flux density (*PPFD*, with units of µmol m^–2^ s^–1^) or, more appropriately for plant growth, integrated on a daily basis as the daily light integral (*DLI*, with units of mol m^–2^ d^–1^). PAR is commonly divided into three wavebands: blue (B; 400–500 nm), green (G; 500–600 nm), and red (R; 600–700 nm) light. Each waveband independently and interactively regulates plant growth and development (Fig. 1b) with additional contributions coming from UV (280–400 nm), near-infrared (NIR) or far-red (FR; 700–750 nm) wavebands^6,13,14^. Of these, B and FR light strongly regulate plant morphology and development, altering characteristics such as leaf area, stem length, and flowering^15–18^. Although there can be benefits to decreasing incoming solar radiation in some cases (e.g., reducing water consumption or soil temperature), adoption of this hybrid field will rely on being broadly applicable by transmitting as much PAR as possible, particularly in temperate climates^4,19–21^. In some agrivoltaic approaches, G light has been considered to have less impact on plant growth because of low absorption by chlorophyll and carotenoids, causing it to be a target wavelength for absorption^20,22–24^. However, as shown in Fig. 1b, metrics such as relative action and quantum yield show that plants utilize G light quite efficiently in photosynthesis^6,25^. Green light is particularly useful in penetrating deeper into leaves under high-light conditions and reaching leaves that are shaded by others^26,27^. Thus, spectral manipulation by absorbing specific wavebands within PAR (Fig. 1c) will alter plant growth and yield, which will vary among species and cultivars of plants. Allowing growers to maintain control of how plants grow and develop while still providing the benefits of agrivoltaics will be essential to enable future widespread adoption.

Recent efforts have been made to introduce and improve visible transparency of PV cells, either broadband or with selectively absorbing materials, to increase application to a greater number of surfaces^28–31^. We can evaluate these approaches in the context of agrivoltaics, from spatially segmenting opaque solar cells to wavelength-selective active materials^20–22,24,32–35^. This is a key distinction because they offer fundamentally different theoretical limits as a function of transparency. The theoretical limit of a spatially segmented cell is 0% at 100% transparency and around 21% for wavelength-selective transparent photovoltaics (TPVs) at 100% transparency. Agrivoltaic implementations based on opaque or visibly absorbing materials will always have a tradeoff between power generation and plant productivity. As a result, agrivoltaic studies have primarily focused on the financial tradeoff of power generation over reduced crop yield^12,21,23^. While financially the tradeoff is of slight benefit, notable losses in crop yield will limit the implementation of agrivoltaics for locations or seasons with limited or moderate PAR availability. Each study also typically focused on only one type of plant under an exceedingly small area of PVs, limiting the translatability as to how these different agrivoltaic approaches will affect a variety of species.

In this work, we evaluate the effects of TPVs with neutral shading and with wavelength-selective shading on three diverse and highly representative and commercially important species of plants grown in greenhouses: the culinary herb basil (*Ocimum basilicum*), the flowering ornamental petunia (*Petunia* × *hybrida*), and the fruit-bearing tomato (*Solanum lycopersicum*). Emphasis is placed around the VIS and NIR edge to better understand where it is appropriate to absorb light for TPV without affecting plant growth and development. We show that overall *DLI* has particularly important ramifications for growth, yield, and morphology across species and that weighted *DLI* further impacts these metrics, indicating wavelength-specific phenomena should be studied further. This work will enable the design of agrivoltaic approaches to ensure greater compatibility with existing agricultural infrastructure across a large range of crop types and in regions with different solar availability.

## Results

**Figure 2:**
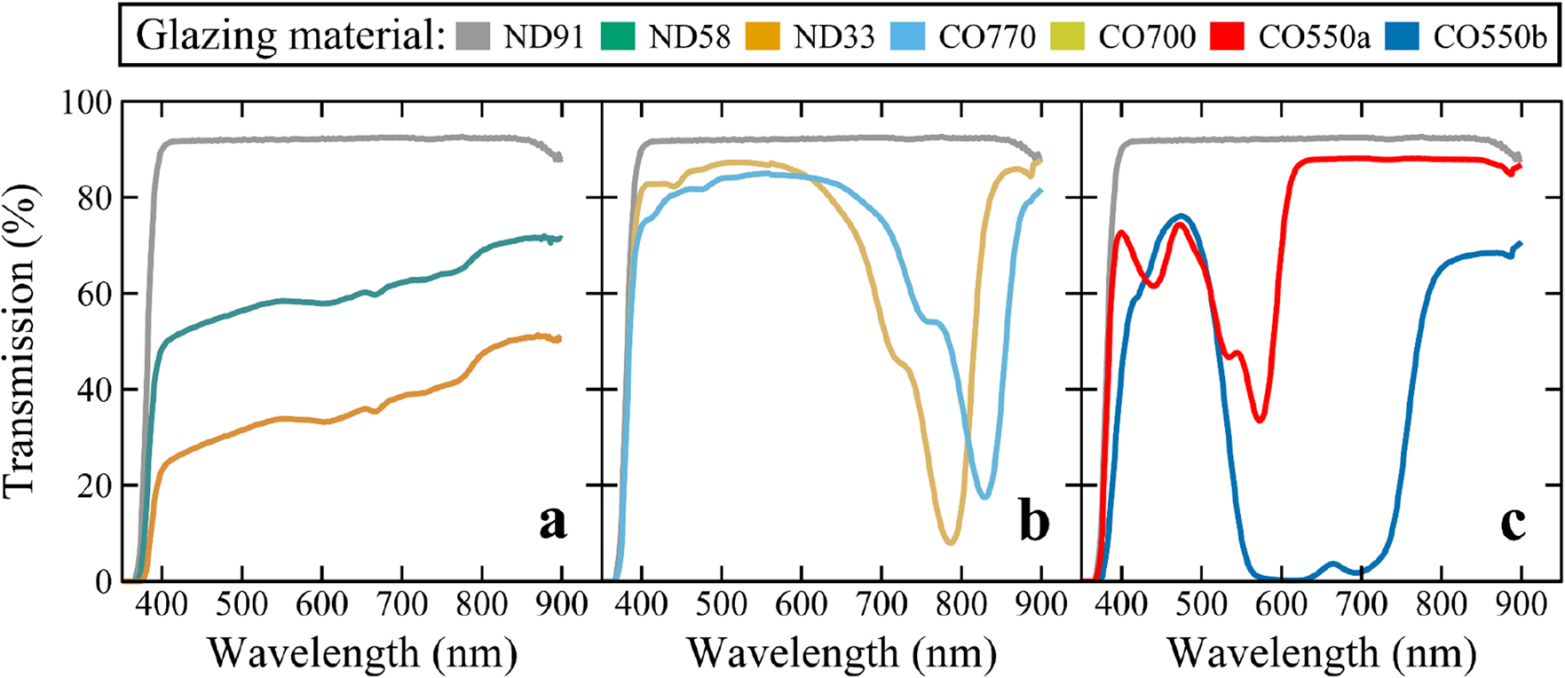
Transmitted photon spectra of glazing materials. Measurements were made inside chambers covered with various glazing materials with different spectral transmissions on a clear day around solar noon. (**a**) ND91 (91% transmission) (repeated in **b** and **c**), ND58 (58% transmission), and ND33 (33% transmission) were acrylic sheets with different photon transmissions. (**b**) CO770, CO700, and (**c**) CO550a, and CO550b were experimental photoselective glazing materials with different wavelength transmission cutoffs in the photosynthetically active radiation (PAR; photons between 400 and 700 nm) and the near-infrared (NIR) wavebands.

We constructed seven ventilated chambers (each a roof area of ~ 0.96 m^2^) that were each covered with a different experimental TPV glazing material (Figure S1, S2). We provided three neutral-shading treatments to quantify the effects of PAR loss on crop production: 91% (ND91), 58% (ND58), and 33% (ND33) transmittance across PAR (Fig. 2a). We also developed four wavelength-selective treatments based on luminescent solar concentrator (LSC) molecule platforms^36^ designed with different cutoffs to determine how removing specific wavebands and overall *DLI* affect plant growth, development, and yield (Fig. 2b and c): two in the NIR (CO700 and CO770) and two in the PAR wavebands (CO550a and CO550b). We note that the absorption profiles of these panels have the same absorption profile of complete transparent luminescent solar concentrator modules but do not have PV strips mounted around the edge.

**Figure 3:**
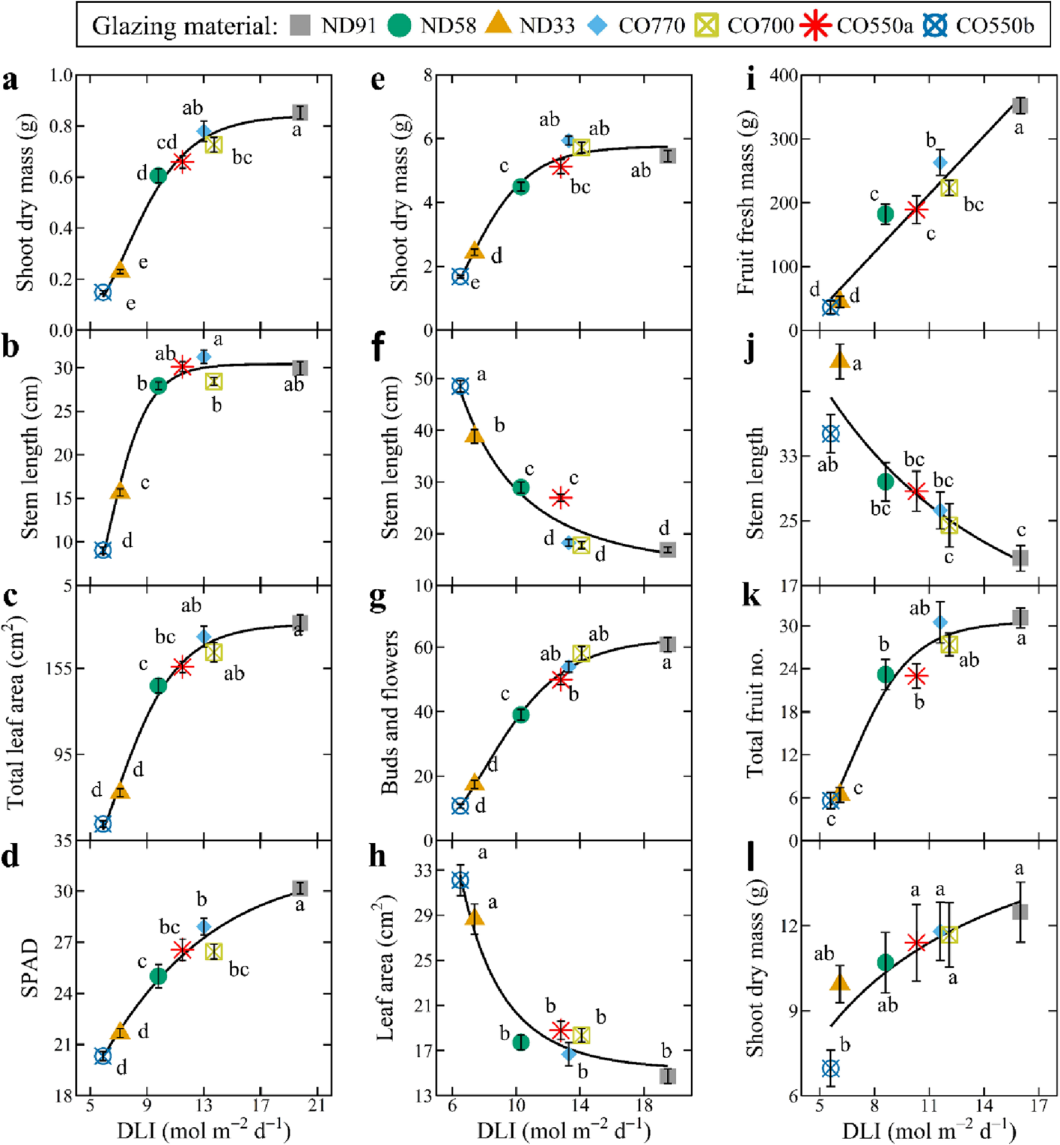
Selected growth response regressions from basil, petunia, and tomato. Growth parameters of basil (**a**–**d**), petunia (**e**–**h**), and tomato (**i**–**l**) under various glazing materials with different spectral transmissions. The transmission spectra for the different glazing materials are given in Fig. 2. Shoot dry mass for basil and tomato refers to both leaves and stems. Data represent means ± SE with ten samples. Means with different letters are significant according to Tukey’s honestly significant difference test (*P* < 0.05). Regression equations for basil, petunia, and tomato are presented in Tables S1–S3, respectively.

Figure 3 depicts selected growth attributes of basil, petunia, and tomato as a function of the *DLI* transmitted through the different glazing treatments. Each yield or quality parameter is plotted as a function of the average treatment *DLI*, since *DLI* is strongly correlated with plant growth and yield for many greenhouse crops^37^; regression equations are displayed in Tables S1–S3. We selected these attributes because they are important to the yield and quality of each crop and vary among crop types. Basil yield reflects the biomass accumulation of leaves and stems whereas stem length, leaf size, and color are quality parameters (Fig. 3a–d). While it is important to quantify the biomass accumulation of petunia, floriculture crops derive more of their marketability from aesthetic qualities such as stem length (preference for compactness) and number of flowers (floral display) (Fig. 3e–h). Unlike basil and petunia, tomato yield reflects fruit fresh mass and number (Fig. 3i–l).

### Basil growth

**Figure 4:**
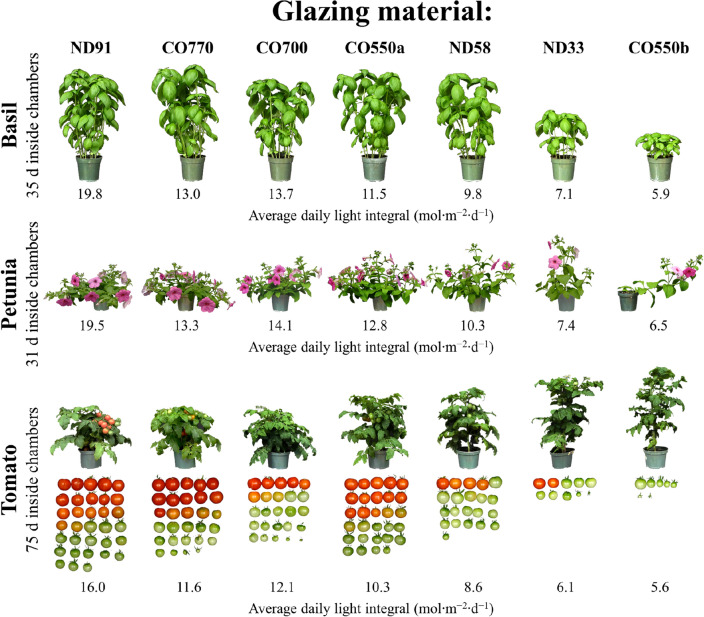
Representative plants under each glazing material. Photos of basil, petunia, and tomato plants representative of those grown under various experimental glazing materials on 16 June 2020, 21 July 2020, and 13 Oct. 2020, respectively. The transmission spectra for the different glazing materials are given in Fig. 2.

Basil growth and development parameters measured were highly correlated with the average transmission *DLI*. We characterized basil yield as the average shoot dry mass per plant (leaves plus stems). Regardless of the spectral transmission differences among treatments, there was a sigmoidal relationship between the yield of basil and the average *DLI* (Fig. 3a; equation Table S3), where yield increased linearly when the *DLI* was between ~ 6 and ~ 12 mol m^–2^ d^–1^. Yield did not increase much further when the *DLI* exceeded ~ 12 mol m^–2^ d^–1^. Basil yield was similar when grown under the ND91, CO770, and CO700 treatments, corresponding to treatments with a transmitted *DLI* typically ≥ 12 mol m^–2^ d^–1^ (Table S4). Basil grown between a *DLI* of ~ 10 and ~ 14 mol m^–2^ d^–1^ (i.e., CO770, CO700, and CO550a) had statistically similar stem lengths compared to the ND91 treatment (*DLI* =  ~ 20 mol m^–2^ d^–1^) but produced fewer nodes and thinner stems at the substrate surface (Fig. 3b; Table S4). Elongation and narrowing of basil stems (Fig. 4) caused plants to become progressively less upright compared to the ND91 treatment, which could negatively affect basil consumer appeal or ease of production (i.e., harvest and shipping). In addition to yield, leaf morphology is important to basil cultivation. Similar to basil yield and stem morphology, leaf morphology was correlated with treatment *DLI* (Table S1). Basil leaf length, width, and total surface area were similar when the average *DLI* was ≥ 12 mol m^–2^ d^–1^, which typically occurred in the ND91, CO770, and CO700 treatments (Fig. 3c; Table S4). However, the relative chlorophyll content (*SPAD*) of basil decreased with any amount of shading, resulting in leaves with a lighter green color (Fig. 3d). Reduced pigmentation could negatively affect the aesthetic appeal of basil products. In summation, when *DLI* was ≥ 12 mol m^–2^ d^–1^, basil yield was statistically similar across treatments but came at the consequence of altered stem and leaf morphology.

### Petunia growth

We characterized petunia yield as the average shoot dry mass per plant (leaves plus stems), and regardless of the spectral transmission differences among treatments, there was a sigmoidal relationship between biomass and the average *DLI* (Fig. 3e; equations in Table S2). Yield increased linearly when the *DLI* was between ~ 6 and ~ 12 mol m^–2^ d^–1^. When the *DLI* exceeded ~ 12 mol m^–2^ d^–1^, the yield response was at or near saturation. Thus, petunia yield was similar when grown under the ND91, CO770, CO700, and CO550a treatments when the transmitted *DLI* was typically ≥ 12 mol m^–2^ d^–1^ (Table S5). While shoot biomass is not inconsequential to floriculture crops, floriferousness, time to flower, and overall canopy size can have a larger role in the marketability of a crop. Petunia grown under a *DLI* > 13 mol m^–2^ d^–1^ (i.e., CO770 and CO700) had statistically similar central stem lengths compared to the ND91 treatment (DLI =  ~ 20 mol m^–2^ d^–1^) (Fig. 3f; Table S2). As seen in Fig. 4, when the *DLI* was < 13 mol m^–2^ d^–1^, apical dominance increased in a dose-dependent manner, to the point that lateral branching was completely inhibited in the lowest *DLI* treatment (CO550b, *DLI* =  ~ 7 mol m^–2^ d^–1^). Petunia lateral branch length was statistically similar under treatments with *DLI*s between ~ 10 and ~ 20 mol m^–2^ d^–1^, but petunia lateral branches were significantly longer under the CO550a (*DLI* = 12.8 mol m^–2^ d^–1^) treatment compared to the ND91 treatment. This suggested the CO550a photoselective material that removed many of the B and G photons from transmission increased extension growth. However, the CO550a treatment did not comparably increase leaf area, and petunia that received a *DLI* between ~ 12 and 20 mol m^–2^ d^–1^ had a statistically similar individual leaf size (Fig. 3h). The time to first open flower after transplant was statistically similar for petunia in the ND91, ND58, CO770, CO700, and CO550a treatments, which all had an average *DLI* > 7 mol m^–2^ d^–1^ (Table S5). When *DLI* was ≤ 7 mol m^–2^ d^–1^, petunia flowered ~ 3 days later than those in the ND91 treatment. Petunia produced fewer total flowers when the *DLI* was < 12 mol m^–2^ d^–1^ (Fig. 3g).

### Tomato growth

As a fruiting crop, tomato usually has a longer production time than basil or petunia. We characterized tomato yield as the total fresh mass of all fruits (ripe and unripe) per plant at a single destructive harvest. While most growth parameters for basil and petunia were best described as sigmoidal functions of *DLI*, tomato yield increased linearly with *DLI* and never approached an upper asymptote (Table S6). This indicated that any decrease in PAR transmission negatively influenced yield, and even the coverings with the highest PAR transmission (CO770 and CO700) had 25% and 37% less yield than the ND91 treatment (Fig. 3i). Total number of fruit and fruit dry mass in these three treatments were similar, indicating a decrease in *DLI* decreased fruit size, delayed fruit ripening, or both (Fig. 3k). As such, tomato in the CO770 and CO700 treatments had 52% and 74% fewer ripe fruit at harvest than the ND91 treatment, respectively (Fig. 3i). Tomato leaf morphology was not influenced by the average *DLI* or specific absorption bandwidths. However, tomato stem length increased and stem diameter decreased with decreasing *DLI* (Fig. 3j, 4). As a result, tomato required physical support to remain upright under low *DLI* conditions. The time to first open flower after transplant was statistically similar for tomato in the ND91, ND58, CO770, CO700, and CO550a treatments, which all had an average *DLI* ≥ 6 mol m^–2^ d^–1^ (Table S6). When *DLI* was < 6 mol m^–2^ d^–1^, tomato flowered ~ 14 days later than those in the ND91 treatment.

## Discussion

### Quantum units in agrivoltaics

The field of agrivoltaics is relatively new and being pursued by several interested disciplines in engineering and plant sciences. In this work, we first describe plant responses to *DLI* because it is a quantum unit that drives photosynthesis, is highly correlated with crop yield and quality, and responses have been characterized in a wide range of greenhouse crops^9,38^. Radiometric units (Watts or Joules) that integrate over a wider waveband of electromagnetic radiation are still important to the field of agrivoltaics because they can better describe the effect of building-integrated (BIPV) panels on the microclimate around plants (e.g., air temperature) or plant process such as transpiration that can ultimately influence crop growth.

Definitions for *PPFD* and *DLI* assume any photon with a wavelength between 400 and 700 nm equally powers photosynthesis (i.e., it has the same quantum yield). However, photons can have different quantum efficiencies (the yellow line in Fig. 1b) based on their relative action and leaf absorption^6,25^. Thus, a weighted description of PAR was created to give a more accurate representation of the instantaneous photosynthetic rate based on the spectral distribution of a light source, which is termed yield photon flux density (*YPFD*)^39^. In this case, *YPFD* is not restricted to just PAR; photons < 400 nm and > 700 nm are included, although their efficacy decreases rapidly as photon wavelengths decrease below 400 nm and increase above 700 nm. Recently, there has been a proposed definition change of PAR to consider photons between 400 and 750 nm as photosynthetically active^13,14^, which has been termed extended PAR or ePAR. ePAR is relevant to the current study because whether TPVs begin to cutoff around 700 nm or closer to 750 nm could impact plant growth and energy generation. Regardless of which waveband is used to describe the photometric transmission of a photovoltaic material, reporting at least one of these plant-centric parameters is required to characterize the plant environment appropriately and to make plant growth and yield comparisons among treatments and studies meaningful. While meaningful differences between *DLI*, *eDLI*, and *YPFD* as predictor variables for several basil growth parameters were not observed in the current study, large transmission differences may have attenuated the effect (Supplementary Section 1, Figure S3). Further research into the most applicable quantum unit is still required in the field of agrivoltaics.

### Crop growth and yield

Crop yield (crop biomass per unit area) and quality (e.g., metrics of aesthetics or nutritional density) influence revenue generated from horticultural crops whether grown in a field or controlled environment such as a greenhouse. While yield has a simple mathematical definition, crop quality is subjective and can consider consumer preferences for, and interactions with, an agricultural product. Examples include crop nutrition and flavor and physical qualities that make a crop easier to manage, harvest, ship, or market. Ideally, agrivoltaic systems would have no negative effect on crop yield or quality while creating passive income through electrical generation. An inescapable challenge for designing and using agrivoltaic systems is accounting for the many different crops and growing systems used in plant-based agriculture. The diversity of uses for the same land area should emphasize the need for flexible agrivoltaic systems, since each crop, and even diversity within a crop species, could have specific tolerances to *DLI* reductions and/or removed wavebands of radiation.

To strive towards broad agricultural application of BIPV cover, namely one that would have negligible impact on the yield and quality of different crops, we grew three economically important greenhouse crops that offered diverse comparisons among plants primarily grown for their leaves and stems (basil), flowers (petunia), and fruits (tomato). Generally, basil and petunia are commercially grown under moderate *DLIs*, while light is usually maximized for fruiting crops such as tomato^38^. However, in regions in which light is seasonally limiting, such as > 35°N or S latitude, most commercial greenhouse growers cannot tolerate more than slight reductions in the *DLI*, even for crops such as basil and petunia.

**Figure 5:**
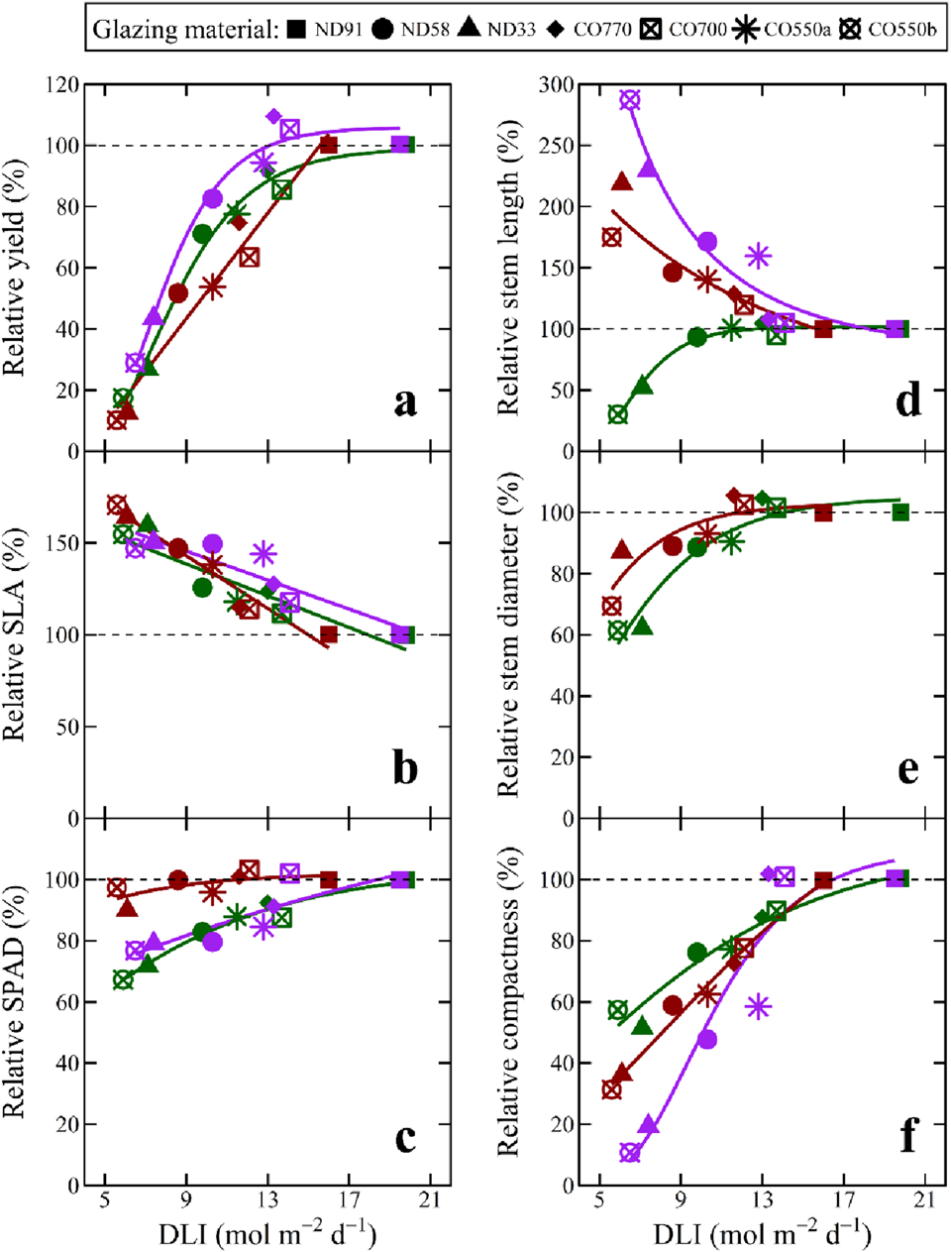
Relative growth response of basil, petunia, and tomato. Relative growth of basil (green symbols and regression lines), petunia (purple symbols and regression lines), and tomato (red symbols and regression lines) under various glazing materials with different spectral characteristics. The transmission spectra for the different glazing materials are given in Fig. 2. Each growth parameter is relative to the average value of the control treatment according to species and represents the average of ten samples. (**a**) Relative yield refers to basil and petunia shoot dry mass (leaves and stems) and tomato fresh fruit mass. (**b**) Specific leaf area (*SLA*) was calculated by dividing leaf area (cm^2^) by leaf mass. (**c**) *SPAD* reflects the relative chlorophyll concentration of leaves. (**d**) Stem length was measured from the substrate surface to the apical meristem. (**e**) Stem diameter was measured at the substrate surface. (**f**) Compactness was calculated by dividing the total above-ground dry mass (**g**) by stem length (cm).

For broad comparison, we plotted relative growth parameters for the three greenhouse crops as a function of the average *DLI* in each treatment (Fig. 5). Data were made relative to the average value of the control treatment for each growth parameter and species. This facilitates more direct response comparisons for the three representative crops commonly produced by greenhouse growers in terms of growth and yield (Fig. 5a), as well as leaf (Fig. 5b,c), stem (Fig. 5d,e), and plant (Fig. 5f) morphological responses. The three crops generally responded similarly to *DLI*, with two notable exceptions: (1) relative yield approached or reached a saturating *DLI* for basil and petunia, but not for tomato; (2) stem length of basil increased with *DLI* until a saturating value, whereas it decreased with *DLI* for petunia and tomato.

Direct comparisons between our results and past agrivoltaic studies are challenging because many of those studies reported shading factors or percentage roof cover of experimental BIPV materials, but actual light conditions would depend on geographic location and time of year, among other factors. For example, lettuce grown under PV harvesters that decreased *PPFD* by as much as 50% caused limited yield reductions in some studies^12,20,33–35,40–42^. In contrast, other studies reported that basil, spinach, lettuce, and arugula yield decreased under PV harvesters that decreased *PPFD* by 25–60%^23,41,42^. Reporting common quantum units and *DLI*, not just reduction in quantum flux, would aid in comparing studies. Many of these studies were conducted in the summer with very high incident *DLIs*, which may have resulted in relatively little yield loss. However, during winter greenhouse conditions, when *DLIs* may be at or below 10 mol m^–2^ d^–1^, any shading may reduce yields and thus a decrease in *DLI* may not be tolerable. Inconsistencies among studies as well as seasonal and geographic differences in solar radiation attest to the challenges of providing consistent shading tolerances for non-fruiting crops. Nonetheless, Touil et al.^12^ concluded that up to 25% shading was generally tolerable to crops, but this would still depend on location and crops grown^12^. In addition to measuring and reporting quantum units, the use of benchmark plant species would help advance agrivoltaic development.

Figure 5a indicates a near-saturating *DLI* for production of high-quality basil and petunia is ~ 12 mol m^–2^ d^–1^, which is consistent with previous studies on sweet basil and petunia, as well as other floriculture crops including impatiens (*Impatiens wallerana*), begonia (*Begonia* × *semperflorens-cultorum*), and ageratum (*Ageratum houstonianum*)^9,19,43,44^. This indicates agrivoltaic panels that decrease the *DLI* by up to 40% (maintaining *DLI* ≥ 12 mol m^–2^ d^–1^) can be useful for greenhouse systems during late spring and summer. However, when the ambient solar *DLI* is lower, such as during the winter and early spring, these reductions would negatively impact growth. Thus, for BIPV panels acting as a permanent greenhouse glazing, acceptable transmissions should heavily consider often suboptimal seasonal conditions for a particular geographical location.

Tomato did not tolerate even modest shading (e.g. a *DLI* < 12 mol m^–2^ d^–1^ or a decrease of 4 mol m^–2^ d^–1^ in the current work) without a decrease in yield or number of ripe fruits at harvest (Fig. 5a), which is consistent with previous literature on tomato and other fruiting crops such as pepper (*Capsicum annuum*) and cucumber (*Cucumis sativus*)^38^. Moreover, it is likely our dwarf tomato cultivar was more tolerant of less light than much larger indeterminate tomato varieties typically grown in greenhouses with *DLIs* > 20 mol m^–2^ d^–1^^45^. Decreased yield and delayed ripening were previously observed as a consequence of BIPV and traditional shading^38,46–50^. Therefore, for tomato and other fruiting crops, BIPV panels used for greenhouse applications should maximize transmission of PAR in temperate regions, but modest decreases in PAR transmission may be tolerable in subtropical, tropical, and especially arid regions.

There was a clear relationship between *DLI* and yield of the three crops studied, yet there is also the potential to manipulate the solar spectrum to increase the yield of at least some species. For example, lettuce, kale (*Brassica oleracea*), geranium (*Pelargonium* × *hortorum*), and snapdragon (*Antirrhinum majus*) had greater shoot dry mass when grown under higher fractions of R and FR photons compared to B and G photons^15–17^. Substituting shorter-wavelength B and G photons for longer wavelength R and FR photons increased leaf area and light interception, which increased biomass accumulation. Thus, semi-transparent PV panels could theoretically be designed to absorb more B and G photons (for greater energy generation) than R and perhaps FR photons (for greater quantum yield) if the altered crop morphology was tolerable. However, for high-light crops such as fruiting vegetables, PV panels with maximal transmission of PAR may be needed, particularly in temperate climates. While the transmission (i.e., *DLI*) differences observed between treatments in the current study likely attenuated spectral effects, more research is needed to elucidate how waveband-selective absorption of B, G, R, and/or FR light influences both energy output and growth of diverse crops.

### Crop morphology and quality

In addition to yield, shading can negatively impact crop quality, but such effects are often not reported in agrivoltaics research. The marketability and quality of many floriculture crops, as well as other ornamentals, is influenced more by their appearance and physical qualities (e.g., flower number and size) than biomass accumulation. Therefore, application of BIPV materials to greenhouses must also consider morphological acclimation, leaf and flower pigmentation, branching, time to flower, and floriferousness of crops. Striking a balance between crop quality, yield, and electrical generation by BIPV panels necessitates a comprehensive approach to crop evaluation in agrivoltaic systems.

#### Leaf morphology

Leaf morphology, pigmentation, and in some cases flavor, are important quality attributes for greenhouse crops sold for their vegetative growth. Figure 5b shows *SLA* was inversely and linearly related to the treatment *DLI* for each species, especially for tomato. Increased *SLA* (i.e., increase leaf surface area at the expense of leaf thickness) is a common plant response to increase light interception^51^. Marrou et al.^34^ suggested that certain plant species that acclimate to shading by increasing their light interception could be more desirable for agrivoltaic systems^34^. However, the thinning of leaves could make them more susceptible to stressors (e.g., pathogens) and physical damage during production and harvest. Leaf pigmentation, which we quantified by relative chlorophyll content (*SPAD*) measurements, increased as a function of the average *DLI* for each crop (Fig. 5c). Among the three crops grown, basil quality could be the most negatively affected by lower chlorophyll concentration (i.e., lighter green color) because its quality is heavily dependent on leaf appearance. In addition to morphology and color, the concentration of flavonoids decreased as *DLI* decreased^52^. Therefore, although leaf morphology and yield of basil, petunia, and tomato were similar when the *DLI* decreased from ~ 20 to ~ 12 mol m^–2^ d^–1^, there were some negative effects on metrics of plant quality.

#### Stem morphology

Stem morphology (e.g., stem length and diameter) can influence the marketability of greenhouse crops. In most cases, commercial growers strive to produce containerized crops that are branched and compact (e.g., short and thick stems) to facilitate shipping and handling. A decrease in *DLI* increased stem elongation of tomato and decreased stem diameter of basil and tomato (Fig. 5d,e). The thinner and longer stems of basil and tomato plants necessitated physical support to remain upright. While this may not influence tomato production because string typically provides support during commercial greenhouse production, it can reduce the quality of potted horticultural crops like basil or increase lodging of agronomic crops like soybean (*Glycine max*), which should remain upright without support. Calculations for compactness (plant mass per unit height) or the ratio of plant height to plant diameter help estimate the space an individual plant occupies during production. Compactness decreased (i.e., each plant occupied more space) as *DLI* decreased in all crop species, suggesting cropping density may need adjustment under some agrivoltaic systems, and this could ultimately influence crop yield (Fig. 5f). Excessive extension growth is usually undesirable for floriculture crops, so the increase in stem elongation and decrease in plant compactness under lower *DLIs* decreased their quality, or would necessitate increased use of plant growth retardants in their management^53^. Similar to leaf morphology, greenhouse crop quality decreased with the transmitted *DLI*, which highlights the nuanced trade-offs that exist between crop yield and quality and the need for BIPV covers with high transmission of PAR.

#### Flowering and fruiting

Commercial greenhouse growers of ornamentals strive to produce crops in the shortest time possible while maintaining at least acceptable plant quality, while growers of fruiting crops seek to maximize yield per unit area and time^54^. We observed delayed flowering of petunia and tomato when the treatment *DLI* was < 7 and 6 mol m^–2^ d^–1^, respectively. Importantly, we started to observe morphological differences and reduced floriferousness when the *DLI* was < 12 mol m^–2^ d^–1^. Our results indicate petunia, and likely other floriculture crops, could tolerate moderate shading without decreasing yield or quality, which makes ornamentals more suitable than fruiting vegetable crops for agrivoltaic systems located in temperate regions. To date, however, few studies have focused on ornamental crops in agrivoltaic systems.

Similar to yield, *DLI* was the predominate factor influencing crop quality parameters. In addition to *DLI*, the light spectrum also influences plant morphology and acclimation responses^15–17^. Responses to light spectrum include: (1) B-light mediated inhibition of cellular expansion and thus leaf area and internode elongation, as well as increased accumulation of secondary metabolites; (2) G-light promotion of *SLA* and stem elongation; and (3) shade-avoidance responses (e.g., increase in extension growth and *SLA*) triggered by a decrease in the ratio of R to FR light. In this study, basil and petunia tolerated some degree of shading without triggering shade-avoidance responses such as thinner leaves and stem elongation, which would decrease crop quality. As with biomass accumulation, the potential exists to develop PVs that modify the solar spectrum to increase crop quality of greenhouse crops. For example, PVs that transmit B light and absorb FR light would likely increase crop compactness, branching, and leaf pigmentation, but could delay flowering of some crops. Additional research is needed to understand how manipulation of the solar spectrum influences quality parameters of various species, as well as qualify trade-offs that may exist with crop growth and biomass accumulation.

### Potential power output of transparent agrivoltaics

Translating semitransparent and TPV modules to plant- and agriculture-based applications requires re-defining key metrics. Typically for TPVs in the window industry, the average visible transmittance (*AVT*) is the most important reported parameter. It is a measure of how much incident solar photon flux passes through the panel or window weighted by the average response of the human eye (i.e., the photopic response). To translate this definition to agrivoltaics systems, we introduce a new metric, the average photosynthetic transmittance (*APT*), which is analogous to *AVT* for the window industry. Replacing the photopic response, we utilize the relative quantum efficiency of plants from McCree^6^, which is defined as the instantaneous CO_2_ consumption rate per photon absorbed that is averaged among 22 varieties of plants and remains the broadest plant quantum efficiency study to date. Thus, *APT* is defined as:

1 $$APT=\frac{\int T\left(\uplambda \right)S\left(\uplambda \right)P\left(\uplambda \right)d\lambda }{\int S\left(\uplambda \right)P\left(\uplambda \right)d\lambda },$$

where *S*(λ) is the AM1.5G photon flux, *T*(λ) is the photon transmittance of the harvester, and *P*(λ) is now the average photosynthetic quantum yield^6,55^. Thus, *APT* is a property of the harvesters placed above plants that ultimately impacts and imparts a particular quantum unit (e.g., *DLI*, *YPFD*, etc.) based on the location and position-dependent solar flux.

We first utilize the definition of *APT* and PAR to define the upper limit for TPVs in agrivoltaics with maximum transparency. For this work, we assume the use of single-junction modules and perfectly sharp cutoffs where the transmittance is 1 between 395 and 715 nm (a wavelength range dictated by setting an *APT* of 95%) and 0 outside that range. The theoretical power conversion efficiency (*PCE*) of PVs is limited by the radiative recombination dark current in the detailed balance limit. This can be calculated via the Shockley ideal diode equation with the dark saturation current as:

2 $${J}_{S}\cong qg\underset{{E}_{G}}{\overset{\infty }{\int }}\frac{{E}^{2}}{\mathrm{exp}\left(\frac{E}{nkT}\right)-1}dE,$$

where $g=2\pi /({c}^{2}{h}^{3})$, *n* is the ideality factor (*n* = 1 in this case), *c* is the speed of light, *h* is Planck’s constant, and *E*_*G*_ is the band gap of the active material in the PV. The thermodynamic single-junction Shockley-Queisser (SQ) limit also assumes one hundred percent internal quantum efficiency, parallel resistance is infinite, series resistance is negligible, only photons with energy equal to or greater than the bandgap are absorbed, and each photon corresponds to exactly one electron._._ For an opaque single-junction PV module, the *PCE* limit is 33.7%, meaning that 33.7% of the total energy from incident solar irradiance can be converted to electricity. The limit is 20.1% for modules that transmit all light between 435 and 670 nm^56^. When expanding the transmission range to 395–715 nm, the resulting theoretical limit is 17.0% (note that the theoretical limits of the absorbers presented in this work can be found in Figure S5 and Table S9). When we apply practical constraints for device operation such as device resistance (assuming 80% voltage limit, 85% maximum external quantum efficiency, and 80% fill factor), the *PCE* limit is around 11% for a device transmitting light between 395 and 715 nm. The thermodynamic limits we define here for standard TPV modules also translate equivalently to the limits for visibly transparent luminescent solar concentrators (TLSCs)^57^. TLSCs are capable of higher *APT* values (~ 90%) because TPV modules are limited by multiple transparent electrodes to an *APT* of ~ 80%; however, TLSCs have lagged behind TPVs in *PCE*^58^.

**Figure 6:**
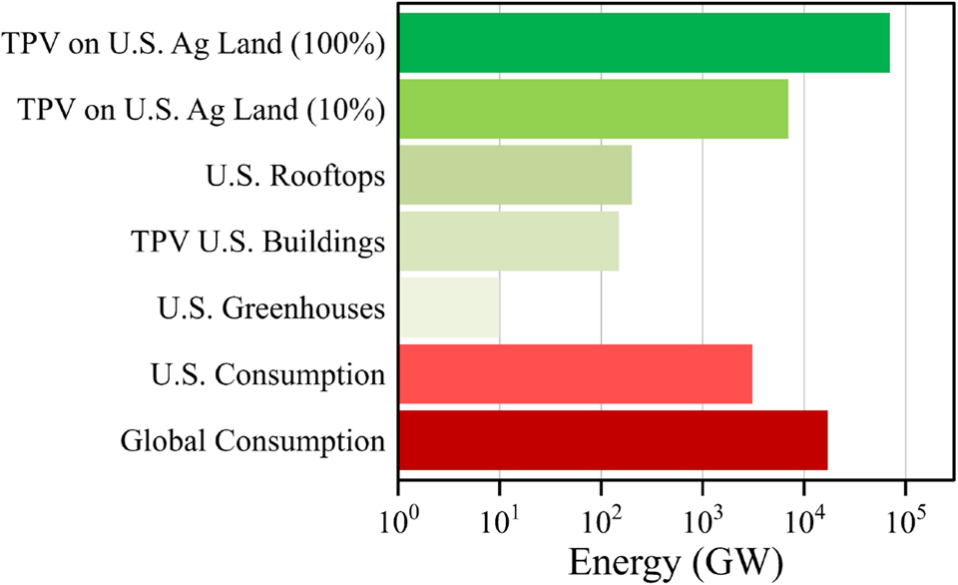
Potential energy output of agrivoltaics. The potential energy output of agrivoltaics on US agricultural land significantly surpasses the energy generation of rooftop solar and other integrated solar approaches. Agrivoltaics with 10%-efficient panels would produce more than double the US energy consumption with only 10% coverage of US agricultural land, and more than quadruple the global energy consumption with 100% coverage of US agricultural land.

We use these limits to project forward and estimate the total potential energy output for transparent agrivoltaics that maintain maximum transparency and minimal plant impact. In the U. S., the total amount of area under protected surfaces (e.g., glass and plastic greenhouses) is approximately 1.1 10^7^ m^2^ for fruit, vegetable, and herb production and 7.0 10^7^ m^2^ for floriculture crop production (for the 17 states surveyed)^59^. Assuming 50% of this covered area is permanent greenhouses, this gives an area of ~ 4.0 10^7^ m^2^. Given that the annual average incident solar insolation across the US is 4.5 kWh m^−2^ d^−1^^60^, we then use benchmark efficiencies up to the calculated limits to show the potential annual energy output (Table S7). Assuming 5%-efficient modules, this translates to 3 TWh annually. While the greenhouse energy output value is modest, it can provide important power generation to cover much of the energy demands of greenhouse operation and produce excess energy in high solar flux regions. This effect could become increasingly important as the use of greenhouses expands to enable growth in regions that are less favorable for plant growth, and indeed could hasten greenhouse adoption. In contrast, the total area of farmland (including pastureland) in the US is 3.6 10^12^ m^2^^61^. Agrivoltaics could further be integrated more widely into fields and farmland, particularly if the tradeoffs between plant productivity and power generation can be minimized with the proposed TPV design approaches. In this case, array support structures that enable operating equipment (e.g., tractors and irrigation systems) to function as necessary would be important. Indeed, such solar installations could be synergistically and simultaneously installed with irrigation systems (and perhaps fertilizer and pesticide solutions too) such that the PV mounting systems double as conduit for subsurface, drip, or spray irrigation. At the theoretical limit, the total output would approach 1.0 10^6^ TWh (~ 3500 quadrillion British thermal units, quads), which is more than the entire energy demand of the U.S. across all sectors. We estimate TPV agrivoltaic panels could reasonably cover upwards of 1–10% of farmland area, translating to a minimum of 3.6 10^10^ m^2^ in this case. The use of 5% efficient TPV modules over 1% of farmland results in 3000 TWh annually, enough energy to account for 75% of the U.S. electricity consumption (~ 4000 TWh), and 10%-efficient TPVs would provide 6000 TWh annually, surpassing the entire electricity consumption^62^. Scaling up to 10% of farmland and utilizing practically achievable 10%-efficient TPVs, the total power output would be 60,000 TWh (~ 200 quads), which would more than double the 27,000 TWh (~ 93 quads) of total power generated in the U.S. from all sources in 2020 (Fig. 6)^62^. Thus, even with minimal use of PAR for solar harvesting there remains exceptional opportunity for power generation in transparent agrivoltaics enabling efficient dual land use that can power the entire country and the world.

## Conclusions

In this work, we have presented an unprecedented and comprehensive approach to determine the applicability of TPV greenhouse glazings for diverse and economically important greenhouse crops. Unique to agrivoltaic greenhouse systems is the demand for growing many different crops continuously throughout the year. Currently, more comprehensive agrivoltaics research is necessary to understand what materials are most applicable to the broad range of crops grown in greenhouses while considering agricultural practices and geographical locations. The current work offers a novel plant-centric focus where TPV materials are investigated to minimize the impact on plant growth, productivity, and yield.

Despite the often-dramatic differences in PV panel photon distribution, panel transmission of *PAR* was the most significant predictor of crop yield and quality. Basil and petunia yield and quality responses were saturated when the average *DLI* was > 12 mol m^–2^ d^–1^, which corresponded to ~ 35–40% shading (~ 60–65% *APT*). This indicates tremendous potential for herbs and floriculture crops in agrivoltaic systems from late spring to early fall when solar irradiance is high. However, the fruiting crop tomato experienced reduced yield with even moderate BIPV shading, and thus *APT* > 65%, is even more strongly desired. Additional studies are necessary to determine a more precise red, far-red, near-infrared cutoff for fruiting crops under a variety of total *DLIs* to provide greater context for more diverse geographical locations. Pushing the absorption edge deeper into the NIR (> 750 nm) increases the transmitted *DLI* and therefore should reduce the impact of TPVs on fruiting crops, resulting in productivity more comparable to the control of single-pane glass. Lastly, this work identifies the need for consistent quantum unit reporting in agrivoltaics to improve study reproducibility and applicability for future agrivoltaic systems, whether building- or field-based. Establishing appropriate *APT*, *DLI*, and wavelength cutoffs for different types of crops is a necessary step towards developing TPVs for a range of truly synergistic agrivoltaic implementations.

## The experimental section

### Preparation of wavelength-selective harvesters (CO550a, CO700, CO770)

2-[2-[2-Chloro-3-[(1,3-dihydro-1,3,3-trimethyl-2H-indol-2-ylidene)ethylidene]-1-cylcohexen-1-yl]-ethenyl]-1,3,3-trimethyl-1H-indolium iodide (IR775-I, Few Chemicals) and 2‐[2‐[2‐chloro‐3‐[2‐(1,3‐dihydro‐3,3‐dimethyl‐1‐ethyl‐2H‐benz[e]indol‐2‐ylidene)ethylidene]‐1‐cylohexen‐1‐yl]‐ethenyl]‐3,3‐dimethyl‐1‐ethyl‐1H‐benz[e]indolium iodide (Cy-I, American Dye Source). Cy-I and IR775-Cl were mixed with potassium tetrakis(pentafluorophenyl)borate (K-TPFB) as described elsewhere^63^ to create Cy-TPFB (CO770) and IR775-TPFB (CO700). Lumogen F Red 305 (CO550a) was purchased from BASF. The dyes were dissolved in ethanol and mixed with Shandon mounting media (CAS#9990435, Thermo Fisher Scientific) in a solution to mountant volume ratio of 1:2. This mixture was drop-cast onto acrylic sheets and allowed to dry in a fume hood for 6 h. The dried harvesters were transferred into a glovebox under nitrogen. A layer of epoxy (KATIOBOND) was applied to the outer edge of the film, and a glass sheet was placed on top of the epoxy layer. The epoxy was treated with UV light until cured, and the active area of the harvester was covered with a mask to reduce UV exposure prior to the study.

### Preparation of wavelength-selective harvester of CO550b

Copper (II) Pthalocyanine (CuPc) (CO550b) films were grown on acrylic panes in a custom thermal evaporator from Angstrom Engineering by evaporating powdered CuPc (Sigma Aldrich) in a tungsten boat. The acrylic was mounted on a rotation stage. The film was grown at a rate of 2 Å/s to a thickness of 5000 Å at room temperature and a pressure of less than 3 × 10^–6^ torr.

### Preparation of neutral density treatment

The Neutral Density Gray panels were purchased from ePlastics. Two pieces were stacked on top of each other to achieve the ND33 treatment.

### Chamber construction

Seven chambers were constructed, each covered by different luminescent solar concentrator harvesters or ND panels. These chambers were placed on the benches of a research greenhouse (Figure S1, S2). Each chamber was 92 cm wide, 98 cm long, and had a total volume of 0.66 m^3^ (Figure S1a). Chamber frames were constructed of polyvinyl chloride (PVC) pipe and enclosed on the four sides perpendicular to the base with opaque 1.3 cm thick insulation board to ensure light reaching plants passed solely through the panels. Moreover, we painted the inside of the insulation boards with flat white paint to increase light scattering. Each experimental chamber was constantly ventilated by heavily perforating the north-facing wall and installing one 120 V, 3.1 m^3^ min^−1^ fan (Axial 1238, AC Infinity Inc., City of Industry, CA) on the south-facing wall, which is in line with the research greenhouse airflow (Figure S1b). Chamber roof frames were fabricated from steel angle-bar and pitched 20 degrees toward the south to maximize sunlight transmission to plants inside.

### Environmental sensing

Quantum sensors (LI-190SA; LI-COR, Inc., Lincoln, NE, or SQ-500; Apogee Instruments, Inc.) measured instantaneous *PPFD* and were located on the north-facing wall of each chamber and maintained level with the top of the plant canopies (Figure S1b). One aspirated thermocouple (Type E; Omega Engineering, Inc., Stamford, CT) per chamber measured air temperature. A CR-1000 datalogger (Campbell Scientific, Logan, UT) and AM16/32B multiplexer (Campbell Scientific) sampled instantaneous air temperature and *PPFD* measurements every minute and recorded hourly averages. Average daily temperature and *DLI*) were calculated and recorded (Figure S4).

### Greenhouse environment

Each chamber was in an east-to-west oriented glass-glazed research greenhouse at Michigan State University (42.7° N/84.5° W) on individual aluminum benches. A greenhouse environmental control system (Integro 725; Priva North America, Vineland Station, ON, Canada) regulated the air temperature at a set point of 21 °C. Radiant steam heating, roof vents, exhaust fans, and an evaporative cooling pad regulated air temperature. The air temperature inside the experimental chambers averaged 25, 27, and 24 °C for basil, petunia, and tomato, respectively, while air temperature differences between each chamber varied by a maximum of 2 °C (Figure S4a–c; Table S8).

### Basil seedling culture

Basil seeds (Johnny’s Selected Seeds, Winslow, ME) were sowed directly into round 4-in pots (473 mL) filled with a greenhouse media consisting of 70% peat moss, 21% perlite, and 9% vermiculite (Suremix; Michigan Grower Products, Inc., Galesburg, MI) and placed inside experimental chambers on 12 May 2020. Each 4-in pot contained seven basil plants. Irrigation was provided as needed with a solution consisting of reverse osmosis water supplemented with 13N–1.3P–12.5 K water-soluble fertilizer that contained (in mg L^−1^) 125 N, 13 P, 120 K, 77 Ca, 19 Mg, 1.7 Fe, 0.4 Cu, and Zn, 0.8 Mn, 0.2 B and Mo (MSU Orchid RO Water Special; GreenCare Fertilizers, Inc., Kankakee, IL).

### Petunia and tomato seedling culture

Petunia seeds (Harris Seeds Co., Rochester, NY) were sown in a controlled-environment growth room on 29 May 2020 into 288-cell (8 mL individual cell volume) plug trays filled with a propagation mix consisting of 50% of greenhouse media mentioned previously (Suremix; Michigan Grower Products, Inc.) and 50% vermiculite by volume. Seeds of tomato, a dwarf variety, (Park Seed Co., Hodges, SC) were sown in the same growth room on 13 July 2020 into 128-cell (17.5 mL individual cell volume) plug trays filled with the same propagation mix as petunia. Petunia and tomato germinated under a 10 h and 18 h photoperiod, respectively, at a constant 23 °C and a *PPFD* of 175 µmol m^–2^ s^–1^. Sole source electrical lighting was provided with a white light-emitting diode (LED) fixture (RAY22; Fluence, Austin, TX). Transparent plastic humidity domes covered the germinating seedlings until cotyledon emergence (6 d). Seedlings were irrigated as needed with a solution of deionized water, hydroponic water-soluble fertilizer (12N–1.7P–13.3 K RO Hydro FeED, JR Peters, Inc, Allentown, PA), and magnesium sulfate (Epsom salt, Pennington Seed Inc., Madison GA) that provided the following nutrients (in mg L^–1^): 125 N, 18 P, 138 K, 73 Ca, 49 Mg, 37 S, 1.6 Fe, 0.5 Mn, 0.4 Zn, 0.2 B and Cu, and 0.01 Mo. Seedling stock solution pH and electrical conductivity were measured upon formulation with a handheld meter (HI9814; Hanna Instruments, Woonsocket, RI) and adjusted to a pH of 5.8 and electrical conductivity of 1.2 mS cm^–1^.

### Mature crop culture

Ten pots of basil, petunia, and tomato were randomly placed into each chamber at a density of 10 pots m^−2^ until ready for harvest. Each pot was filled with the same peat-based greenhouse media described for basil seedling culture. Basil grew inside the chambers for 35 d until harvest on 16 June 2020. Petunia were transplanted into 4.5-in round pots on 20 June 2020, and grew inside chambers until 21 Jul. 2020 (31 d) when all plant had at least one fully open flower. Tomato seedlings were transplanted into 4.5-in round posts on 30 Jul. 2020. Tomato seedlings were transplanted when they developed a good root system (17 d) and grew inside experimental chambers for 75 d until harvest on 13 Oct. 2020, which is when plants in the ND91 chamber had ripe fruit. Basil, petunia, and tomato were irrigated as needed with the identical solution described in the basil seedling culture.

### Plant data collection

At harvest, the following data were measured for both basil, petunia, and tomato: stem length (from the substrate to the apical meristem); fresh and dry above-ground biomass using scales (GR-200 and GX-1000; A&D Store, Inc., Wood Dale, IL); the length, width, area, and relative chlorophyll content of the youngest fully expanded leaf using a ruler, leaf area meter (LI-3100 Area Meter; LI-COR, Inc.), and relative chlorophyll content with hand-held meter (MC-100; Apogee Instruments, Inc., Logan, UT). Basil, petunia, and tomato fresh samples were dried for at least four days in parchment bags at 60 °C in a drying oven (Blue M, Blue Island, IL) before dry mass was measured. In addition to the measurements taken for both species, independent measurements were taken for basil, petunia, and tomato. For basil, we also measured the total number of expanded leaves, expanded nodes, and branches > 5 cm long; total leaf area of all expanded leaves; stem diameter at substrate level with a digital caliper (41101 DigiMax; Wiha Switzerland, Monticello, MN, USA); and fresh and dry mass of just the leaves or stems. Because each basil pot contained seven plants, we selected three plants from each pot that excluded the two tallest and two shortest plants and averaged their growth metrics. For petunia we counted the time to visible bud and flower since seed sow, number of branches > 10 cm, inflorescence, and nodes under the first flower; and measured the longest lateral branch. For tomato, we also measured fruit number (ripe and unripe); fruit fresh and dry mass; and the date on which the first flower had fully reflexed petals. Specific leaf area (*SLA*) was calculated by dividing the youngest fully expanded leaf's area by its dry mass, and plant compactness by dividing its above-ground dry mass (excluding fruit for tomato) by its stem length according to Burnett et al.^64^ Two-dimensional projected canopy area (*PCA*) was recorded for each tomato plant with an overhead photograph and analyzed in ImageJ software (http://imagej.nih.gov/ij).

### Experimental design and statistics

The experiment was organized as a completely randomized design where treatments (7 levels) and plants were assigned to random chambers (experimental units) inside the research greenhouse. Data were analyzed in R software (Version 4.0.3, The R Foundation, Vienna, Austria) using analysis of variance (ANOVA) and Tukey's honestly significant difference test at *α* = 0.05. Regression analysis comparing basil and tomato growth parameters (i.e., dry mass, stem length, or leaf area) as a function of the average *DLI* was first evaluated as a linear or quadratic function, but often trends appeared sigmoidal, in which the following Gompertz function was used:

3 $$y=a\mathrm{exp}\left(-b {c}^{x}\right),$$

where *y* = response variable (growth parameter), *a* = asymptote, *b* = displacement on the x-axis, *c* = growth rate, and *x* = predictor variable (*DLI*). The Gompertz function is an asymmetrical logistic function where the right-hand portion of the curve approaches the upper asymptote more gradually than the left-hand portion approaches the lower asymptote. Past studies have used the Gompertz function to describe the growth of biological organisms as a function of time^65^ and plant growth responses as a function of cumulative thermal energy^66^ and *DLI*^67^. The Gompertz function was selected over the symmetrical logistic function because curves visually fit the data better and typically had higher *R*^*2*^ values.

### Ethical approval

All experiments were performed with commercially available seed. Methodology was in compliance with institutional, national, and international guidelines and legislation.

## Supplementary Information

Supplementary Information.

## Data Availability

The datasets used and/or analyzed in this manuscript are available from the corresponding author on reasonable request by E-mail.
