# Supplementary material for: Designing plant–transparent agrivoltaics
Source: Sci Rep. 2023 Feb 2;13:1903. doi: 10.1038/s41598-023-28484-5 (PMC9895072; doi:10.1038/s41598-023-28484-5)
Supplement: Supplementary file 1 — Supplementary Information. [file 41598_2023_28484_MOESM1_ESM.docx]

Supplemental Information

**Designing Plant-Transparent Agrivoltaics**

*Eric J. Stallknecht, Christopher K. Herrera, Thomas D. Sharkey, Richard R. Lunt, and Erik S. Runkle**


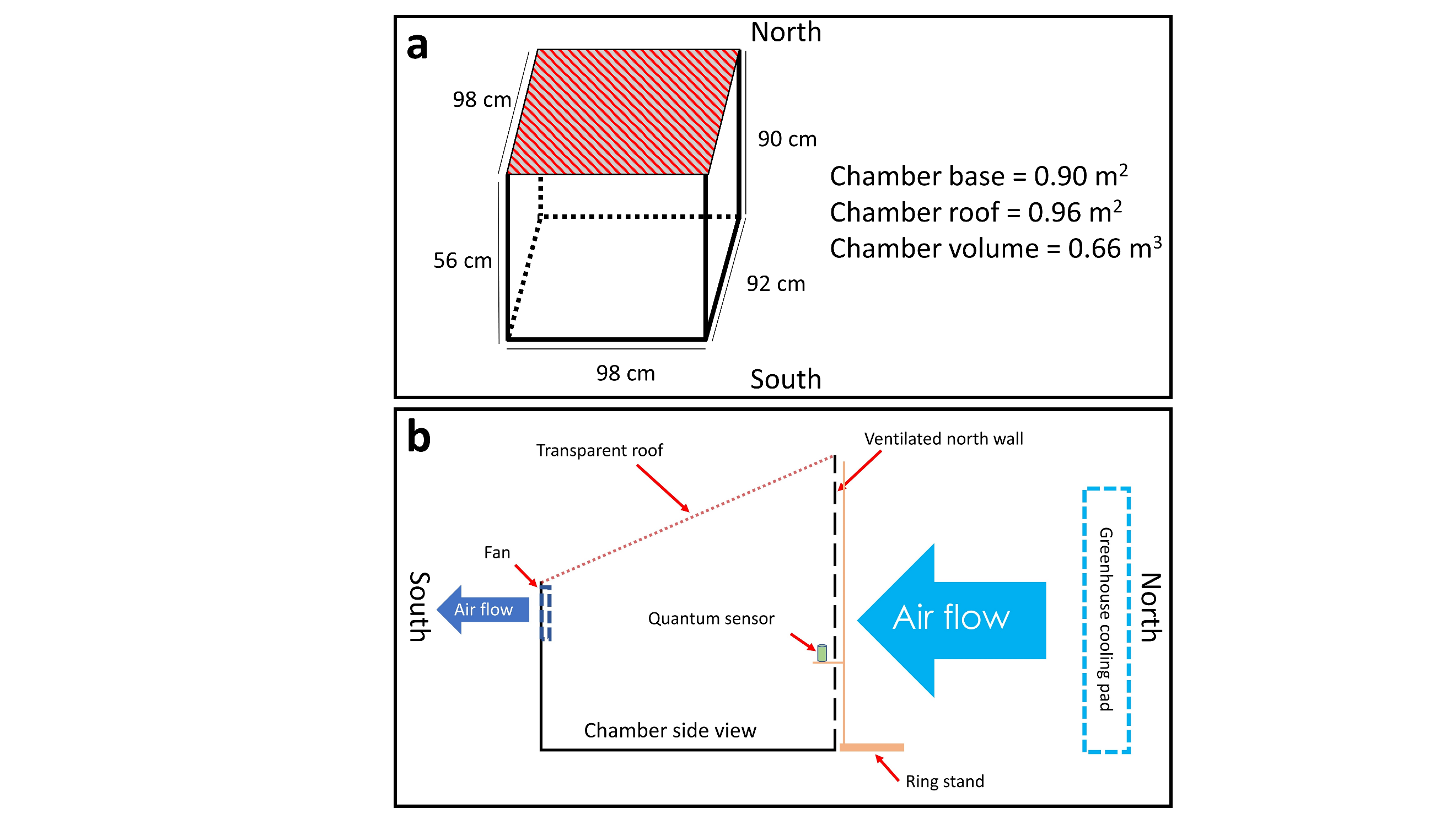


**Supplementary Figure 1: Chamber design and dimension.** a) Experimental chamber dimensions and b) greenhouse orientation schematic.


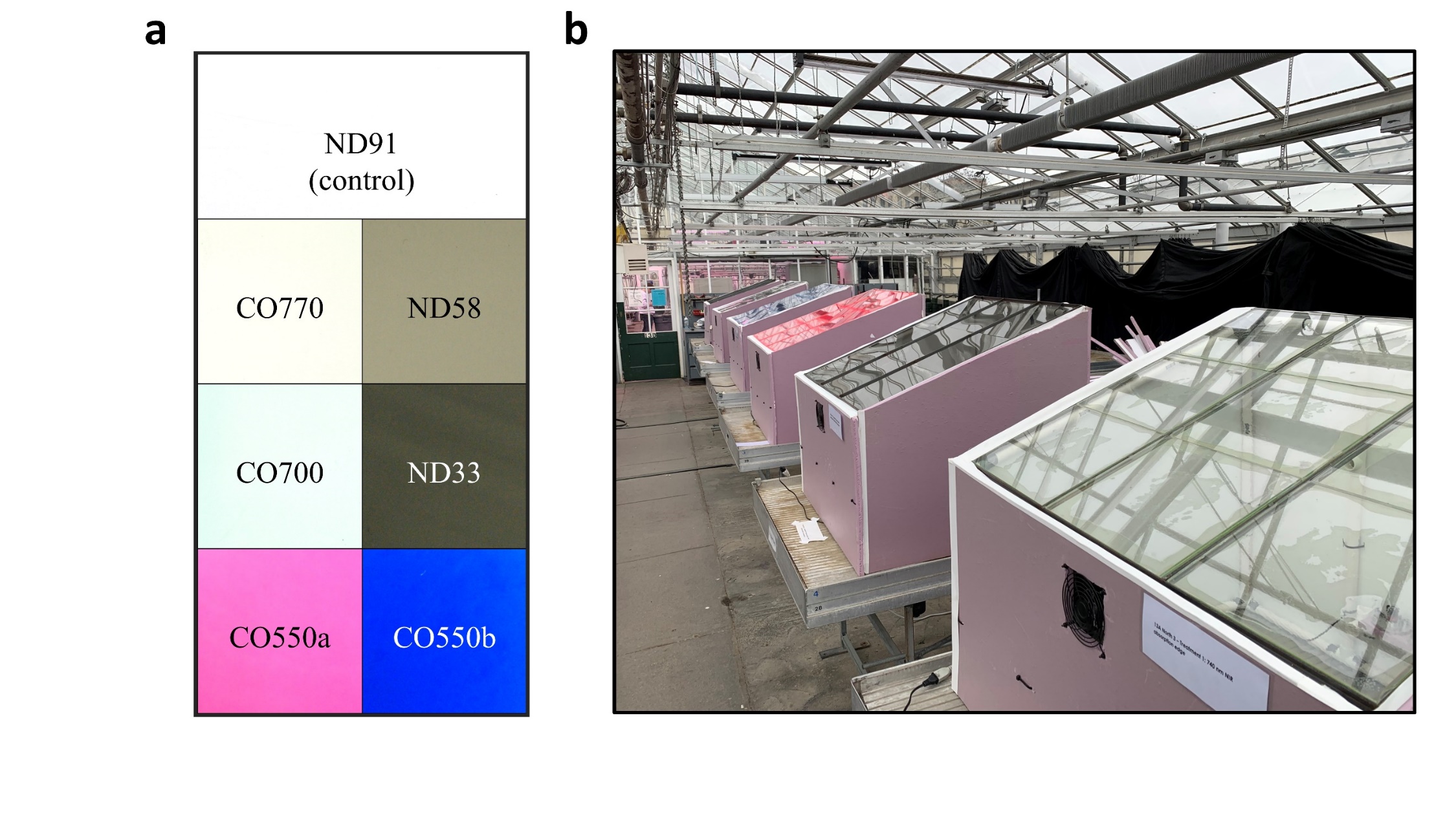


**Supplementary Figure 2: Visual representation of glazing materials and chambers inside the greenhouse.** Photographs of various materials used for experimentation. a) ND91 (91% transmission), ND58 (58% transmission), and ND33 (33% transmission) were acrylic sheets with different *PPFD* transmissions. CO770, CO700, and CO550b were experimental photoselective glazing materials with different transmission cutoffs. The CO550a glazing contained a fluorophore dye that absorbed blue and green photons and fluoresced red and far-red photons. b) Experimental chambers were roofed with various experimental glazing materials inside a Michigan State University research greenhouse.

**Supplementary section 1. Assessing crop growth with various quantum units**

To determine the applicability of additional plant-centric quantum units like *YPFD* and *eDLI* to predict crop growth, we transformed our treatment *DLIs* to reflect the *YPFD* and ePAR using spectroradiometer measurements. Then, we compared the transformed predictor variables to a subset of our basil growth metrics (shoot dry mass, stem length, total leaf area, and relative chlorophyll content) to determine if one parameter was preferable for agrivoltaic reporting (Figure S1).

The sigmoidal curves shifted to lower mol m^–2^ d^–1^ values when *DLI* was transformed into *YPFD*, a result of quantum efficiencies less than one for most PAR wavelengths. Curves shifted to higher mol m^–2^ d^–1^ values when transformed into *eDLI*, the result of integrating photons from a wider waveband. Each regression using the transformed predictor variables had an almost identical sigmoidal shape and similar ability to function as the *DLI* predictor variable for the entire subset of basil growth metrics selected. The observed critical illumination threshold (the mol m^–2^ d^–1^ required to grow a crop with similar yield and quality to the ND91 treatment) for basil was an average *DLI* of ~12 mol m^–2^ d^–1^. After transformation, the critical illumination threshold became ~10 mol m^–2^ d^–1^ and ~14 mol m^–2^ d^–1^ for *YPFD* and *eDLI*, respectively. Generally, these values appropriately reflect the imperfect quantum efficiency of *YPFD* (lower values) and the extended waveband integral of *eDLI* (higher values). However, the magnitude of change upon the x-axis will inevitably depend on the transmission spectra and transformation. For instance, because the CO700 treatment absorbed a large fraction of the FR photons, its *DLI* and *eDLI* should be closer (lower magnitude of change) compared to a neutral-density TPV that does not absorb FR photon (higher magnitude of change). Regardless, even though the magnitude of transformation existed between our treatments, due to the larger magnitude of difference between treatment *DLIs*, the effect of the x-axis transformations were likely minimized.

These results suggest that *YPFD* and *eDLI* were comparable to the traditional, unweighted *DLI* definition to predict plant yield and growth, despite spectral differences being utilized in the agrivoltaic design. As such, it may be beneficial to the field of agrivoltaics to use *eDLI* (the integration of photons between 400 and 750 nm) as the benchmark quantum unit as explained by Zhen and Bugbee (2020a and 2020b).^[1,2]^ Yet, regardless of the quantum unit, these results emphasize the necessity for the use of at least one quantum unit in agrivoltaic reporting but leaves room for further research into a common quantum metric that best describes PV panel transmission relative to crops grown underneath them. Furthermore, the use of *DLI* transformations such as these may be more important and meaningful when the average panel transmissions are more similar.


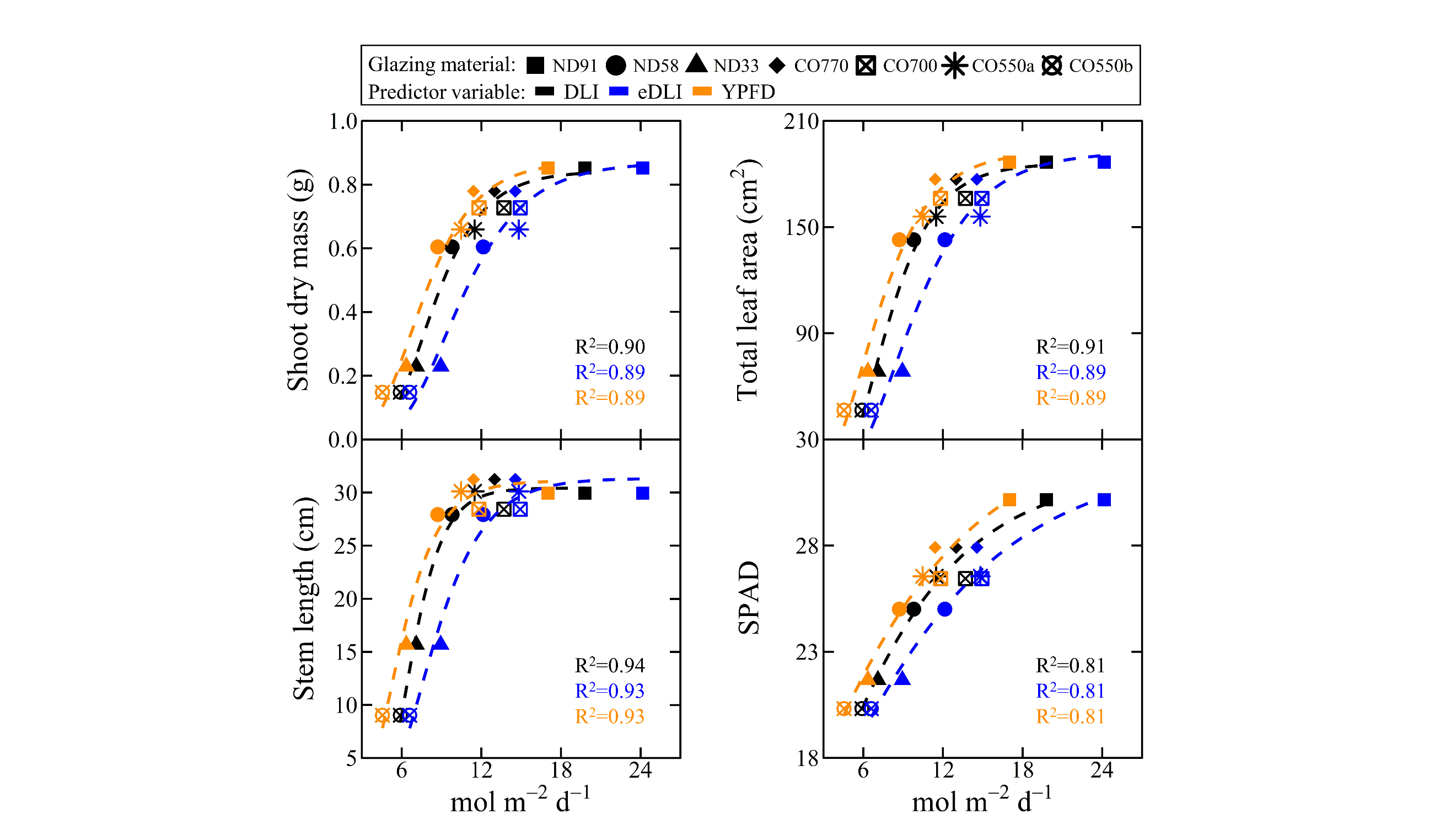


**Supplementary Figure 3: Basil growth parameters as a function of three predictor variables.**Growth parameters of basil as a function of daily light integral (*DLI*; 400–700 nm), extended daily light integral (*eDLI*; 400–750 nm) and average photosynthetic transmission (*APT*; synonymous with yield photon flux density according to McCree, 1972). Average treatment *DLI* was recorded throughout the duration of the experiment and then transformed into *eDLI* and *APT* using spectroradiometric data. Typically, *APT* values are lower and *eDLI* values are greater than *DLI*.
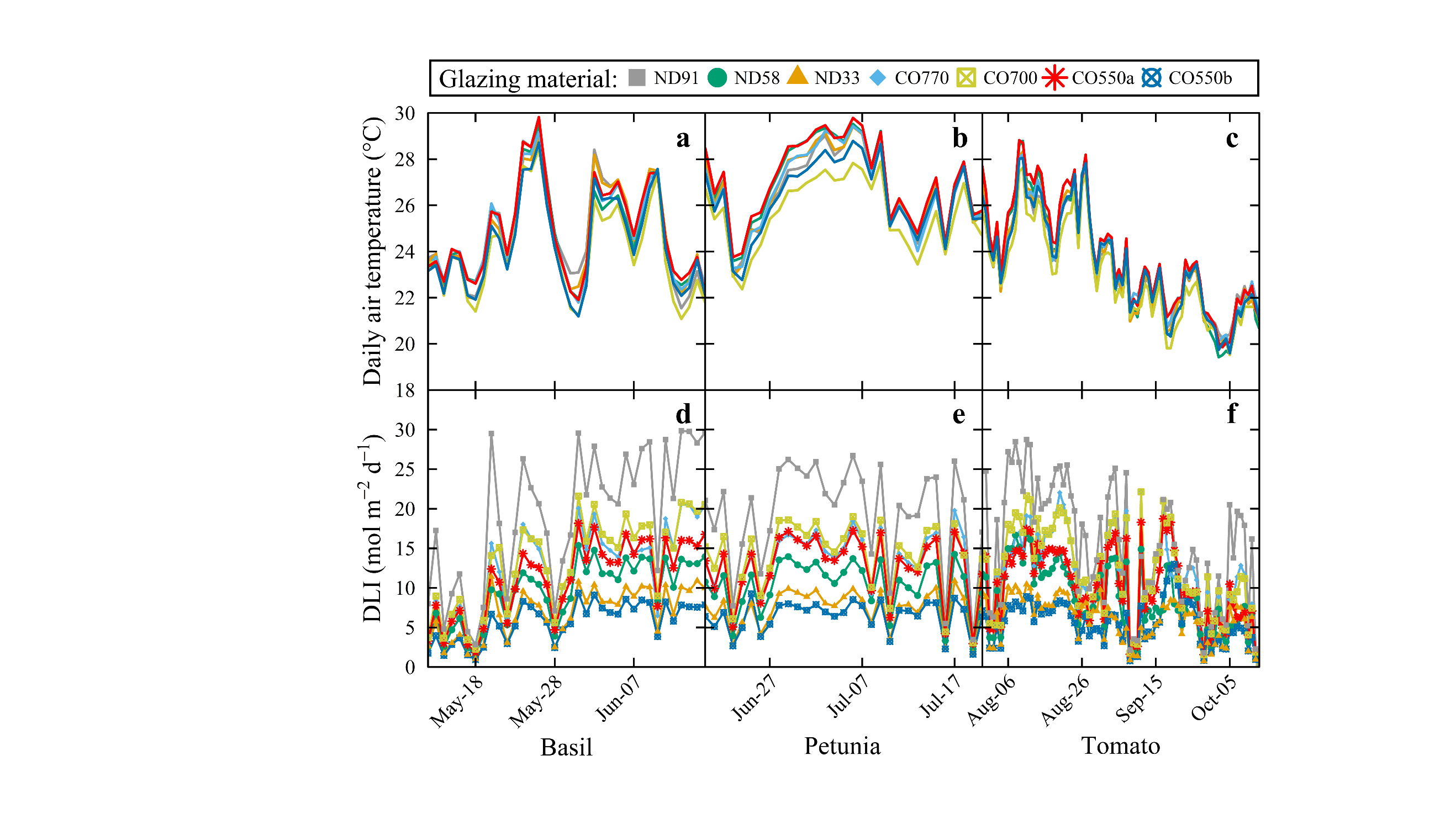


**Supplementary Figure 4: Chamber environmental conditions time series.** a–c) Average daily air temperature (ºC), and d–f) daily light integral (*DLI*; 400–700 nm; mol m^–2^ d^–1^) inside each chamber roofed with experimental glazing materials for basil (a and d), petunia (b and e), and tomato (c and f). ND91 (91% transmission), ND58 (58% transmission), and ND33 (33% transmission) were acrylic sheets with different *PPFD* transmissions. CO770, CO700, and CO550b were experimental photoselective glazing materials with different transmission cutoffs. The CO550a glazing contained a fluorophore dye that absorbed blue and green photons and fluoresced red and far-red photons. Instantaneous photosynthetic photon flux density (400–700 nm) was measured at canopy height every minute and integrated over 24 h to calculate *DLI*. Differences between chamber *DLIs* reflect differences between experimental glazing transmissions that ranged from 91% (ND91) to 34% (VIS550b) of incident sunlight directly above the chambers.

**Supplementary Figure 5: Transmittance Spectrum of idealized NIR absorber.** The transmittance spectra of CO700 and CO770 were combined in the NIR region to create a more ideal absorption profile for TPVs. The UV region shares the same values as the CO700 covering. The calculated average PAR transmittance and theoretical power conversion efficiency are listed in Supplementary Table 9.

**Supplementary Table 1: Basil growth response regressions.** Regression analysis of basil growth parameters (y) as a function of daily light integral (*DLI*; mol m^–2^ d^–1^; 400–700 nm) (x). The regression type with the highest *R^2^* value was selected to describe the data. Sigmoidal regressions were always Gompertz functions (Equation 2). Specific leaf area (SLA) was calculated by dividing leaf area (cm^2^) by leaf mass (g). Compactness was calculated by dividing the total above-ground dry mass by stem length (cm).

| Growth parameter | Regression type | Equation | R^2^ |
| --- | --- | --- | --- |
| Leaf fresh mass (g) | Sigmoidal | $y=6.90 *exp(-20.8*{0.647}^{X})$ | 0.92 |
| Leaf dry mass (g) | Sigmoidal | $y=0.507*exp(-14.6*{0.701}^{X})$ | 0.90 |
| Stem fresh mass (g) | Sigmoidal | $y=4.22*exp(-81.4*{0.556}^{X})$ | 0.90 |
| Stem dry mass (g) | Sigmoidal | $y=0.267*exp(-43.4*{0.615}^{X})$ | 0.89 |
| Total leaf area (cm^2^) | Sigmoidal | $y=186*exp(-15.9*{0.670}^{X})$ | 0.91 |
| Individual leaf area (cm^2^) | Quadratic | $y=5.43-0.190x^{2}-12.7$ | 0.83 |
| Leaf length (cm) | Sigmoidal | $y=9.80*exp(-9.91*{0.575}^{X})$ | 0.72 |
| *SPAD* | Sigmoidal | $y=32.5*exp(-1.11*{0.855}^{X})$ | 0.81 |
| *SLA* (cm^2^ g^−1^) | Sigmoidal | $y=276*exp(1.13*{0.909}^{X})$ | 0.72 |
| Stem length (cm) | Sigmoidal | $y=30.4*exp(-55.2*{0.529}^{X})$ | 0.94 |
| Stem diameter (mm) | Sigmoidal | $y=3.61*exp(-3.76*{0.735}^{X})$ | 0.85 |
| Node no. | Sigmoidal | $y=5.24*exp(-1.60*{0.829}^{X})$ | 0.81 |
| Branch no. | Linear | $y=0.0554x-0.302$ | 0.27 |
| Compactness (g cm^−1^) | Sigmoidal | $y=0.0316*exp(-1.84*{0.860}^{X})$ | 0.78 |

**Supplementary Table 2: Petunia growth response regressions**. Regression analysis of petunia growth parameters (*y)* as a function of daily light integral (*DLI*; mol m^–2^ d^–1^; 400–700 nm) (*x*). The regression type with the highest R^2^ value was selected to describe the data. Sigmoidal regressions were always Gompertz functions (Equation 2). Time to flower was recorded as the number of days from seed sow to first open flower. Specific leaf area (*SLA*) is calculated by dividing leaflet area (cm^2^) by leaf mass (g). Compactness is calculated by dividing the total above-ground dry mass by central stem length (cm). Sigmoidal regressions were Gompertz functions (Equation 2).

| Growth parameter | Regression type | Equation | *R^2^* |
| --- | --- | --- | --- |
| Shoot fresh mass (g) | Sigmoidal | $y=93.3*exp(-53.2*{0.565}^{X})$ | 0.88 |
| Shoot dry mass (g) | Sigmoidal | $y=5.76*exp(-26.1*{0.629}^{X})$ | 0.90 |
| Leaf length (cm) | Sigmoidal | $y=6.83*exp(2.80*{0.710}^{X})$ | 0.54 |
| Leaf width (cm) | Sigmoidal | $y=3.51*exp(1.47*{0.858}^{X})$ | 0.60 |
| Leaf area (cm^2^) | Sigmoidal | $y=15.3*exp(4.58*{0.758}^{X})$ | 0.78 |
| *SPAD* | Linear | $y=0.622x+19.5$ | 0.47 |
| *SLA* (cm^2^ g^−1^) | Linear | $y=-6.64x+304$ | 0.55 |
| Central stem length (cm) | Sigmoidal | $y=13.6*exp(3.31*{0.862}^{X})$ | 0.89 |
| Longest branch (cm) | Sigmoidal | $y=30.2*exp(-1.13e^{4}*{0.196}^{X})$ | 0.79 |
| Branch no. | Sigmoidal | $y=8.81*exp(-61.9*{0.603}^{X})$ | 0.91 |
| Compactness (g cm^−1^) | Sigmoidal | $y=0.357*exp(-22.2*{0.718}^{X})$ | 0.84 |
| Time to visible bud (d) | Sigmoidal | $y=15.0*exp(54.5*{0.373}^{X})$ | 0.38 |
| Time to flower (d) | Sigmoidal | $y=24.6*exp(1.53*{0.649}^{X})$ | 0.34 |
| Total inflorescence no. | Sigmoidal | $y=62.8*exp(-17.6*{0.703}^{X})$ | 0.93 |
| Nodes under first flower no. | Sigmoidal | $y=7.51*exp(104*{0.407}^{X})$ | 0.43 |

**Supplementary Table 3: Tomato growth response regressions**. Regression analysis for various tomato growth parameters (*y*) as a function of daily light integral (*DLI*; mol m^–2^ d^–1^; 400–700 nm) (*x*). The regression type with the highest *R^2^* value was selected to describe the data. Sigmoidal regressions were always Gompertz functions (Equation 2). Projected canopy area (PCA) was measured perpendicular to the top of the canopy. Time to flower was recorded as the number of days from seed sow to first open floret. Specific leaf area (SLA) was calculated by dividing leaflet area (cm^2^) by leaf mass (g). Compound leaflets from a representative leaf of each sample were excised from the petiole before the area and dry mass were measured. Compactness was calculated by dividing the total above-ground dry mass by central stem length (cm). Sigmoidal regressions were Gompertz functions (Equation 2).

| Growth parameter | Regression type | Equation | *R^2^* |
| --- | --- | --- | --- |
| Shoot fresh mass (g) | Sigmoidal | $y=116*exp(-4.92e^{6}*{0.0566}^{X})$ | 0.33 |
| Shoot dry mass (g) | Rectangular hyperbola | $y=\frac{17.9*x}{6.25+x}$ | 0.19 |
| fruit fresh mass (g) | Linear | $y=30.4x-121$ | 0.80 |
| Fruit dry mass (g) | Sigmoidal | $y=16.1*exp(-13.7*{0.704}^{X})$ | 0.75 |
| Total fruit no. | Sigmoidal | $y=30.6*exp(-34.0*{0.593}^{X})$ | 0.75 |
| Ripe fruit no. | Linear | $y=1.53x-9.30$ | 0.64 |
| Unripe fruit no. | Sigmoidal | $y=19.1*exp(-292*{0.392}^{X})$ | 0.41 |
| Leaf length (cm) | - | $\mathrm{NS}$ | - |
| Leaf width (cm) | - | $\mathrm{NS}$ | - |
| Leaflet area (cm^2^) | Linear | $y=3.19x+98.0$ | 0.14 |
| *SPAD* | Linear | $y=0.252x+31.5$ | 0.07 |
| *PCA* (cm^2^) | Sigmoidal | $y=6.38*exp(4.47*{0.980}^{X})$ | 0.40 |
| *SLA* (cm^2^ g^−1^) | Linear | $y=-23.2x+671$ | 0.67 |
| Stem length (cm) | Sigmoidal | $y=6.09*exp(2.42*{0.957}^{X})$ | 0.45 |
| Stem diameter (mm) | Sigmoidal | $y=10.9*exp(-2.74*{0.680}^{X})$ | 0.41 |
| Compactness (g cm^−1^) | Linear | $y=0.0409x-0.0150$ | 0.64 |
| Flower time (d) | Sigmoidal | $y=40.4*exp(11.0*{0.529}^{X})$ | 0.68 |

**Supplementary Table 4: Basil growth under various glazing materials.** Materials had differing photosynthetic photon flux densities (*PPFDs*; 400–700 nm) transmissions and photon distributions. ND91 (91% transmission), ND58 (58% transmission), and ND33 (33% transmission) were acrylic sheets with different PPFD transmissions. CO770, CO700, and CO550b were experimental photoselective glazing materials with different transmission cutoffs. The CO550a glazing contained a fluorophore dye that absorbed blue and green photons and fluoresced red and far-red photons. Specific leaf area (*SLA*) was calculated by dividing the leaf area (cm^2^) by leaf mass (g) of a representative leaf. Compactness is calculated by dividing the total above-ground dry mass by stem length (cm). Data represent means with ten samples. Means with different letters are significant according to Tukey’s honestly significant difference test (*P*<0.05) and correspond to each row. Darker shaded cells correspond to each parameter’s highest values, and lighter shaded cells the lowest values and reflect pair-wise comparisons.

| Growth parameter | Glazing material | | | | | | |
| --- | --- | --- | --- | --- | --- | --- | --- |
|  | ND91 | ND58 | ND33 | CO770 | CO700 | CO550a | CO550b |
| Leaf fresh mass (g) | 6.8 **a** | 5.5 **c** | 2.3 **d** | 6.7 **a** | 6.5 **ab** | 5.7 **bc** | 1.7 **d** |
| Leaf dry mass  (g) | 0.51 **a** | 0.35 **d** | 0.14 **e** | 0.45 **ab** | 0.43 **bc** | 0.38 **cd** | 0.093 **e** |
| Stem fresh mass (g) | 4.0 **ab** | 3.3 **c** | 1.1 **d** | 4.4 **a** | 4.1 **ab** | 3.7 **bc** | 0.5 **d** |
| Stem dry mass (g) | 0.28 **a** | 0.20 **c** | 0.061 **d** | 0.26 **ab** | 0.23 **bc** | 0.22 **bc** | 0.027 **d** |
| Individual leaf area (cm^2^) | 20 **c** | 23 **bc** | 16 **d** | 27 **a** | 26 **ab** | 24 **b** | 13 **d** |
| Total leaf area (cm^2^) | 186 **a** | 143 **c** | 68 **d** | 177 **ab** | 166 **ab** | 156 **bc** | 47 **d** |
| Leaf length (cm) | 9.1 **b** | 9.9 **a** | 7.5 **c** | 10.0 **a** | 9.8 **ab** | 9.8 **ab** | 7.0 **c** |
| *SPAD* | 30.2 **a** | 25.0 **c** | 21.7 **d** | 27.9 **b** | 26.4 **bc** | 26.6 **bc** | 20.3 **d** |
| *SLA* (cm^2^ g^−1^) | 329 **c** | 414 **b** | 525 **a** | 407 **b** | 368 **bc** | 389 **b** | 509 **a** |
| Stem length (cm) | 29.9 **ab** | 27.9 **b** | 15.7 **c** | 31.2 **a** | 28.5 **b** | 30.1 **ab** | 9.0 **d** |
| Stem diameter (mm) | 3.4 **a** | 3.1 **b** | 2.1 **c** | 3.6 **a** | 3.5 **a** | 3.1 **b** | 2.1 **c** |
| Node no. | 5.2 **a** | 4.3 **b** | 3.4 **c** | 4.5 **b** | 4.3 **b** | 4.4 **b** | 3.1 **c** |
| Branch no. | 0.7 **a** | 0.3 **ab** | 0.0 **b** | 0.6 **a** | 0.4 **ab** | 0.4 **ab** | 0.0 **b** |
| Compactness (g cm^−1^) | 0.029 **a** | 0.022 **d** | 0.015 **e** | 0.25 **bc** | 0.026 **ab** | 0.022 **cd** | 0.016 **e** |

**Supplementary Table 5: Petunia growth response under various glazing materials.** Glazing materials has differing photosynthetic photon flux densities (*PPFDs*; 400–700 nm) transmissions and photon distributions. ND91 (91% transmission), ND58 (58% transmission), and ND33 (33% transmission) were acrylic sheets with different *PPFD* transmissions. CO770, CO700, and CO550b were experimental photoselective glazing materials with different transmission cutoffs. The CO550a glazing contained a fluorophore dye that absorbed blue and green photons and fluoresced red and far-red photons. Time to flower was recorded as the number of days from seed sow to first open flower. Specific leaf area (*SLA*) is calculated by dividing leaflet area (cm^2^) by leaf mass (g). Compactness is calculated by dividing the total above-ground dry mass by central stem length (cm). Data represent means with ten samples. Means with different letters are significant according to Tukey’s honestly significant difference test (*P*<0.05) and correspond to each row. Darker shaded cells correspond to each parameter’s highest values, and lighter shaded cells the lowest values and reflect pair-wise comparisons.

| Growth parameter | Treatment | | | | | | |
| --- | --- | --- | --- | --- | --- | --- | --- |
|  | ND91 | ND58 | ND33 | CO770 | CO700 | CO550a | CO550b |
| Shoot fresh mass (g) | 81.1 **cd** | 74.6 **d** | 43.8 **e** | 102.8 **a** | 97.7 **ab** | 89.3 **bc** | 25.6 **f** |
| Shoot dry mass (g) | 5.5 **ab** | 4.5 **c** | 2.4 **d** | 5.9 **a** | 5.7 **ab** | 5.1 **bc** | 1.7 **e** |
| Leaf length (cm) | 6.9 **b** | 7.4 **b** | 8.6 **a** | 6.7 **b** | 7.2 **b** | 7.3 **b** | 9.2 **a** |
| Leaf width (cm) | 3.6 **c** | 4.3 **bc** | 5.8 **a** | 4.4 **bc** | 4.5 **b** | 4.5 **b** | 6.1 **a** |
| Leaf area (cm^2^) | 14.7 **b** | 17.7 **b** | 28.7 **a** | 16.7 **b** | 18.3 **b** | 18.8 **b** | 32.1 **a** |
| *SPAD* | 30.8 **ab** | 24.5 **d** | 24.4 **d** | 28.1 **bc** | 31.4 **a** | 26.0 **cd** | 23.7 **d** |
| *SLA* (cm^2^ g^−1^) | 168 **d** | 251 **a** | 253 **a** | 215 **bc** | 198 **cd** | 242 **ab** | 247 **a** |
| Central stem length (cm) | 16.9 **d** | 28.9 **c** | 38.8 **b** | 18.2 **d** | 17.8 **d** | 27.0 **c** | 48.6 **a** |
| Longest branch (cm) | 27.3 **bc** | 31.2 **ab** | 23.7 **c** | 29.5 **ab** | 29.5 **ab** | 33.3 **a** | 10.7 **d** |
| Branch no. | 8.3 **ab** | 5.6 **c** | 3.0 **d** | 9.0 **a** | 8.7 **ab** | 7.8 **b** | 0.0 **e** |
| Compactness (g cm^−1^) | 0.33 **a** | 0.16 **b** | 0.06 **c** | 0.33 **a** | 0.33 **a** | 0.19 **b** | 0.04 **c** |
| Time to visible bud (d) | 15.3 **b** | 14.9 **b** | 15.6 **ab** | 14.9 **b** | 15.0 **b** | 14.9 **b** | 16.4 **a** |
| Time to flower (d) | 24.4 **cd** | 25.0 **bd** | 26.4 **ab** | 24.6 **cd** | 25.9 **ac** | 23.9 **d** | 26.9 **a** |
| Inflorescence no. | 60.8 **a** | 39.0 **c** | 17.4 **d** | 53.9 **ab** | 58.1 **ab** | 49.9 **b** | 10.7 **d** |
| Nodes under first flower no. | 7.9 **bd** | 6.9 **cd** | 8.8 **ab** | 7.9 **bd** | 8.2 **bc** | 6.6 **d** | 10.1 **a** |

**Supplementary Table 6: Tomato growth response under various glazing materials.** Glazing materials had differing photosynthetic photon flux densities (*PPFDs*; 400–700 nm) transmissions and photon distributions. ND91 (91% transmission), ND58 (58% transmission), and ND33 (33% transmission) were acrylic sheets with different PPFD transmissions. CO770, CO700, and CO550b were experimental photoselective glazing materials with different transmission cutoffs. The CO550a glazing contained a fluorophore dye that absorbed blue and green photons and fluoresced red and far-red photons. Projected canopy area (*PCA*) was measured perpendicular to the top of the canopy. Time to flower was recorded as the number of days from seed sow to first open floret. Specific leaf area (*SLA*) is calculated by dividing leaflet area (cm^2^) by leaf mass (g). Compound leaflets from a representative leaf of each sample were excised from the petiole before the area, and dry mass was measured. Compactness is calculated by dividing the total above-ground dry mass by central stem length (cm). Data represent means with ten samples. Means with different letters are significant according to Tukey’s honestly significant difference test (*P*<0.05) and correspond to each row. Darker shaded cells correspond to each parameter’s highest values, and lighter shaded cells the lowest values and reflect pair-wise comparisons.

| Growth parameter | Glazing material | | | | | | |
| --- | --- | --- | --- | --- | --- | --- | --- |
|  | ND91 | ND58 | ND33 | CO770 | CO700 | CO550a | CO550b |
| Shoot fresh mass (g) | 119 **a** | 108 **a** | 103 **a** | 116 **a** | 122 **a** | 115 **a** | 70 **b** |
| Shoot dry mass (g) | 12.5 **a** | 10.7 **ab** | 9.9 **ab** | 11.8 **a** | 11.7 **a** | 11.4 **a** | 7.0 **b** |
| Fruit fresh mass (g) | 352 **a** | 182 **c** | 45 **d** | 263 **b** | 223 **bc** | 189 **c** | 36 **d** |
| Fruit dry mass (g) | 15.8 **a** | 9.6 **b** | 1.7 **c** | 13.0 **ab** | 12.3 **ab** | 10.5 **b** | 2.3 **c** |
| Total fruit no. | 31.1 **a** | 23.2 **b** | 6.4 **c** | 30.5 **ab** | 27.4 **ab** | 23.0 **b** | 5.6 **c** |
| Ripe fruit no. | 17.9 **a** | 4.9 **bc** | 0.6 **cd** | 8.5 **b** | 4.7 **bd** | 5.7 **b** | 0 **d** |
| Unripe fruit no. | 13.2 **bc** | 18.3 **ab** | 5.8 **c** | 22.0 a**b** | 22.7 **a** | 17.3 **ab** | 5.6 **c** |
| Leaf length (cm) | 20.7 **a** | 18.7 **ab** | 20.4 **a** | 17.1 **b** | 19.5 **ab** | 19.7 **ab** | 18.3 **ab** |
| Leaf width (cm) | 17.2 | 16.4 | 16.4 | 15.1 | 17.0 | 17.3 | 15.1 |
| Leaflet area (cm^2^) | 159 **a** | 122 **b** | 125 **ab** | 111 **b** | 132 **ab** | 144 **ab** | 114 **b** |
| *SPAD* | 24.7 **ab** | 34.6 **ab** | 31.2 **b** | 35.0 **ab** | 35.8 **a** | 33.3 **ab** | 33.8 **ab** |
| *PCA* (cm^2^) | 177 **c** | 291 **ab** | 382 **a** | 199 **bc** | 189 **c** | 237 **bc** | 293 **ab** |
| *SLA* (cm^2^ g^−1^) | 323 **d** | 372 **bc** | 367 **ab** | 551 **d** | 475 **d** | 446 **c** | 530 **a** |
| Stem length (cm) | 20.4 **c** | 29.8 **bc** | 44.6 **a** | 26.3 **bc** | 24.4 **c** | 28.6 **bc** | 35.7 **ab** |
| Stem diameter (mm) | 10.6 **ab** | 9.4 **b** | 9.2 **b** | 11.2 **a** | 10.9 **ab** | 9.9 **ab** | 7.4 **c** |
| Compactness (g cm^−1^) | 0.63 **a** | 0.46 **bc** | 0.49 **cd** | 0.20 **d** | 0.37 **ab** | 0.40 **b** | 0.23 **d** |
| Flower time (d) | 39.1 **b** | 41.6 **b** | 50.7 **a** | 40.3 **b** | 42.1 **b** | 42.0 **b** | 55.1 **a** |

**Supplementary Table 7: Potential energy output of agrivoltaic systems in the US.** Land-area availability was approximated where agrivoltaic greenhouses account for 50% of covered growing area in the US and 1% of farmland area is covered for agrivoltaics purposes. The average daily solar insolation in the US (4.5kWh/m^2^/day) was used as the incident power on the TPV modules, which were assigned efficiencies based on current and prospective TPV benchmarks.

| TPV Efficiency (%) | Greenhouse energy output (TWh/year) | Farmland energy output (TWh/year) |
| --- | --- | --- |
| 1 | 0.6 | 600 |
| 2 | 1 | 1200 |
| 5 | 3 | 3000 |
| 10 | 6 | 6000 |
| 15 | 10 | 9000 |

**Supplementary Table 8 | Environmental conditions inside chambers.** Average daily air temperature (ºC) and daily light integral (*DLI*; 400–700 nm; mol m^–2^ d^–1^) inside each chamber roofed with experimental glazing materials. ND91 (91% transmission), ND58 (58% transmission), and ND33 (33% transmission) were acrylic sheets with different PPFD transmissions. CO770, CO700, and CO550b experimental photoselective glazing materials with different transmission cutoffs. The CO550a glazing contained a fluorophore dye that absorbed blue and green photons and fluoresced red and far-red photons. Values represent daily averages ± SD for basil, petunia, and tomato grown inside each chamber.

| Glazing material | Air temperature (ºC) | | | *DLI* (mol m^–2^ d^–1^) | | |
| --- | --- | --- | --- | --- | --- | --- |
|  | Basil | Petunia | Tomato | Basil | Petunia | Tomato |
| ND91 | 24.9 ± 2.2 | 26.6 ± 1.8 | 23.5 ± 2.1 | 19.8 ± 9.0 | 19.5 ± 7.0 | 16.0 ± 7.6 |
| ND58 | 24.7 ± 2.0 | 26.9 ± 1.8 | 23.6 ± 2.5 | 9.8 ± 4.2 | 10.3 ± 3.6 | 8.6 ± 4.3 |
| ND33 | 24.8 ± 2.2 | 26.7 ± 1.8 | 23.5 ± 2.3 | 7.1 ± 3.3 | 7.4 ± 2.7 | 6.1 ± 2.8 |
| CO770 | 24.8 ± 2.0 | 26.7 ± 1.8 | 23.6 ± 2.1 | 13.0 ± 5.8 | 13.3 ± 4.7 | 11.6 ± 5.5 |
| CO700 | 24.1 ± 2.0 | 25.7 ± 1.5 | 23.0 ± 2.1 | 13.7 ± 6.0 | 14.1 ± 4.9 | 12.1 ± 5.8 |
| CO550a | 24.9 ± 2.1 | 27.1 ± 1.8 | 23.9 ± 2.4 | 11.5 ± 5.0 | 12.8 ± 4.4 | 10.3 ± 4.8 |
| CO550b | 24.3 ± 2.0 | 26.3 ± 1.6 | 23.5 ± 2.2 | 5.9 ± 2.6 | 6.5 ± 2.2 | 5.6 ± 2.7 |

**Supplementary Table 9: Average photosynthetic transmittance and theoretical power conversion efficiency of greenhouse coverings.** Average photosynthetic transmittance was calculated using Eq. (1). The thermodynamic efficiency of each covering was calculated by treating the absorption profile as the device external quantum efficiency (*EQE*), so that the internal quantum efficiency (*IQE*) is assumed to be 100%. These spectra are then utilized in the detailed balance single junction Shockley Quiesser analysis.^[3,4]^ First they are integrated using the following equation to determine the short-circuit current density ($J_{SC}^{int}$):

$J_{SC}^{int}=\int S\left( \lambda\right)EQE\left( \lambda\right)d\lambda$,

where *S*(*λ*) is the incident solar photon flux. The limits are then calculated via the Schockley ideal diode equation with 1) infinite parallel resistance, 2) zero series resistance, and 3) radiative-limited recombination dark current:

$J_{S}=qg\int_{E_{G}}^{\infty} \frac{E^{2}}{\exp\left(E/nkT\right)-1}dE$,

where *g* = 2π/(*c*^2^*h*^3^), *n* is the ideality factor (*n* = 1), *c* is the speed of light, and *h* is Planck’s constant. For the ND treatments, we assume the absorption spectra ends at an optimal bandgap of ~900nm (1.38eV).^[5]^ The spectra of CO700 and CO770 were also combined to show a more ideal NIR profile for this application (adjusted spectrum in Supplementary Figure 5).

| Glazing material | Average Photosynthetic Transmittance (%) | Theoretical Power Conversion Efficiency (%) |
| --- | --- | --- |
| ND58 | 57.0 | 11.8 |
| ND33 | 32.7 | 19.3 |
| CO770 | 76.3 | 12.5 |
| CO700 | 75.2 | 11.3 |
| CO550a | 64.0 | 9.3 |
| CO550b | 21.8 | 21.4 |
| CO700+CO770 | 71.7 | 15.1 |

**Supplementary Information References**

[1] Zhen, S., Bugbee, B. Steady-state stomatal responses of C_3_ and C_4_ species to blue light fraction: interactions with CO_2_ concentration. *Plant Cell Environ.* **43,** 1259. (2020a).

[2] Zhen, S., Bugbee, B. Far-red photons have equivalent efficiency to traditional photosynthetic photons: implications of redefining photosynthetically active radiation. *Front. Plant Sci.* **11.** (2020b).

[3] Lunt, R. R. Theoretical limits for visibly transparent photovoltaics. *Appl. Phys. Lett.* **101,** 043902. (2012).

[4] Lunt, R. R., Osedach, T. P., Brown, P. Rowehl, R., J. & Bulović, A.V. Practical roadmap and limits to nanostructured photovoltaics. *Adv. Mater.* **23,** 5712. (2011).

[5] Shockley, W. H., Queisser, J. Detailed balance limit of efficiency of *p-n* junction solar cells. *J. Appl. Phy*. **32,** 510. (1961).
